# Effects of Scalable, Wordless, Short, Animated Storytelling Videos on Flu Vaccine Hesitancy in China: Nationwide, Single-Blind, Parallel-Group, Randomized Controlled Trial

**DOI:** 10.2196/66758

**Published:** 2025-08-27

**Authors:** Wenjin Chen, Lirui Jiao, Qiushi Chen, Zhoutao Zheng, Pascal Geldsetzer, Merlin Greuel, Jennifer Gates, Jinghan Zhao, Till Bärnighausen, Maya Adam, Simiao Chen, Chen Wang

**Affiliations:** 1 School of Population Medicine and Public Health, Chinese Academy of Medical Sciences & Peking Union Medical College, Beijing, China; 2 Heidelberg Institute of Global Health, Heidelberg University, Heidelberg, Germany; 3 Department of Health Policy and Management, University of North Carolina at Chapel Hill, Chapel Hill, NC, United States; 4 The Harold and Inge Marcus Department of Industrial and Manufacturing Engineering, The Pennsylvania State University, Pennsylvania, PA, United States; 5 Department of Medicine, Stanford University, California, CA, United States; 6 Department of Global Health and Population, Harvard T.H. Chan School of Public Health, Boston, MA, United States; 7 Icahn School of Medicine, New York, NY, United States; 8 Chinese Academy of Medical Sciences & Peking Union Medical College, Beijing, China; 9 Africa Health Research Institute, KwaZulu-Natal, South Africa; 10 Department of Pediatrics, School of Medicine, Stanford University, Stanford, CA, United States; 11 Center for Digital Health, School of Medicine, Stanford University, Stanford, CA, United States

**Keywords:** influenza, vaccine hesitancy, scalable, health knowledge, attitudes, practice, computer animation, mobile phone

## Abstract

**Background:**

Low influenza vaccination rates in China pose a serious public health threat. The vaccination prevents infection, but widespread vaccine hesitancy remains a significant barrier. Short, animated storytelling videos may help by conveying health messages in an engaging, culturally neutral format that transcends literacy barriers.

**Objective:**

We aim to investigate whether scalable, short, animated storytelling videos, using different storytelling techniques—humor, analogy, and emotion—could reduce influenza vaccine hesitancy among Chinese adults.

**Methods:**

In this single-blind, parallel-group, randomized controlled trial, we recruited adults in China through quota sampling. Participants were randomly assigned to 1 of 3 short, animated storytelling video intervention groups, each using a different storytelling technique (humor, analogy, or emotion) or a control group in a 1:1:1:1 ratio. After watching the video or being assigned to the control group, participants completed the questionnaire. Influenza vaccine hesitancy was compared between each intervention group and the control group, respectively, as well as between different intervention groups, with *P* values adjusted for multiple comparisons.

**Results:**

A total of 12,000 participants met the inclusion criteria. Participants in any scalable animated storytelling video group showed lower hesitancy than controls (mean difference −0.41, 95% CI −0.60 to −0.23; *P*<.001). Specifically, both intervention groups with humor (video A) and analogy (video B) storytelling techniques resulted in significantly lower hesitancy compared to the control group, with mean differences of −0.44 (99.17% CI −0.75 to −0.13; *P*<.001) for video A and −0.55 (99.17% CI −0.86 to −0.24; *P*<.001) for video B. However, the intervention group with emotion video (video C) did not show significant effects compared to the control group, nor were there significant differences compared with the other 2 intervention groups. In subgroup analyses, video A effectively reduced vaccine hesitancy among urban residents and participants from southern and southwestern China. Video B was effective within participants aged 40‐49 years, both sexes, both urban and rural residents, those with a college education or higher, households with an income of CN ¥90,000‐180,000 (the 2021 official exchange rate of CN ¥1=US $0.155 was used for reference, based on World Bank data), and participants from the southwestern region and the western economic belt.

**Conclusions:**

Our study showed that storytelling videos, especially with humor and analogy, reduced hesitancy among Chinese adults. Our results highlight the importance of selecting appropriate narrative strategies for health communication, particularly for vaccine hesitancy across various demographic and regional contexts. Given the scalability, low cost, and high accessibility of short, animated storytelling videos, integrating them into national health campaigns could enhance vaccine uptake and mitigate hesitancy in underserved populations. Future research should explore the long-term impacts of these interventions on vaccine uptake and their adaptability to other preventive health measures.

## Introduction

Influenza is a highly contagious respiratory illness that poses a significant threat to human health. Approximately one billion cases of seasonal influenza are documented each year, including 3 to 5 million severe cases [1]. Caused by the influenza virus, “the flu” results in 290,000 to 650,000 deaths each year worldwide [1]. In low- and middle-income countries, the risk of severe influenza cases is higher than in high-income countries [2]. In 2021, more than 668,200 influenza cases, contributing to 10.72% of the total global cases, occurred in China [3].

Influenza vaccination is highly effective in preventing influenza and reducing morbidity and mortality associated with this disease [4]. Studies have documented the cost-effectiveness of influenza vaccination for improving influenza outcomes [5,6]. Yet vaccine hesitancy, defined by the World Health Organization (WHO) as the “delay in acceptance or refusal of vaccines despite the availability of vaccination services,” presents a major obstacle to achieving adequate vaccination coverage, and the WHO has identified vaccine hesitancy as one of the top 10 threats to global health [7].

In China, influenza vaccination rates remain low, with a recent global meta-analysis reporting flu vaccine coverage of less than 17% in the general population [8]. Furthermore, despite the Chinese Centers for Disease Control and Prevention’s vaccine recommendations for high-risk populations, studies found that vaccination rates were even lower (14.12%) for those with chronic diseases [8]. Even among health care workers in China, less than 1 in 4 are protected by influenza vaccination (23.1% as reported in a recent global meta-analysis) [8]. In China, important contributors to vaccine hesitancy include concerns about the side effects, safety, and efficacy of influenza vaccines [9-11]. Other studies noted inadequate knowledge and lack of trust as potential contributors to vaccine hesitancy [12,13]. Furthermore, vaccine hesitancy toward the influenza vaccine has been associated with vaccine hesitancy toward other critically important vaccines, such as the COVID-19 vaccine. Across various regions in China, people who have hesitated or refused to get the flu vaccine are more likely to hesitate or refuse to get the COVID-19 vaccine [14]. These findings suggest that addressing vaccine hesitancy toward 1 disease could work synergistically toward improving the uptake of other vaccines.

Short, animated storytelling videos are a novel and powerful approach for promoting health behaviors and communicating health messages to the general public [15,16]. A specialized form of entertainment-education, short, animated storytelling videos can convey evidence-based health messages by engaging audiences, simplifying complex information, eliciting emotion, and bridging cultural contexts [17,18]. This wordless, culturally accessible approach was first used to scale health messages rapidly, across cultural groups, during the COVID-19 pandemic [19,20].

China, with its diverse cultural and language subpopulations, poses an interesting challenge to the rapid scaling of effective health messages—a challenge that could be addressed by short, animated storytelling or similar approaches. For example, a prior study conducted in Xi’an, China, documented the significant positive effect of a 12-minute educational video aimed at promoting influenza vaccine uptake among participants older than 60 years of age, living in the capital city of Shaanxi province [21]. Emerging evidence from multiple studies underscores the potential of video-based interventions for increasing vaccine-related knowledge and improving vaccination outcomes [18,22,23]. The feasibility of implementing this approach is also emphasized by its ease of dissemination on social media platforms. Yet, to date, no studies have explored the effect of scalable, wordless, short-form (<4 min) animated storytelling videos on vaccine hesitancy in China. Additionally, there is a need to explore different storytelling techniques within the short, animated storytelling approach, such as humor, analogy, or emotion-driven storylines [16]. By comparing these different narrative techniques, we can identify which strategy most effectively reduces vaccine hesitancy. This evidence will inform not only future short, animated storytelling content design but also broader policy guidance for integrating rapid, scalable, and culturally adaptable media into routine vaccine promotion.

To fill this gap, our study reports on a large-scale, nationwide, single-blind, parallel-group randomized controlled trial that evaluates the effectiveness of 3 short, animated storytelling videos using different storytelling techniques of humor, analogy, and emotion, respectively, in reducing flu vaccine hesitancy. We hypothesize that all 3 short, animated storytelling videos are effective in reducing flu vaccine hesitancy among Chinese adults compared to the control group.

## Methods

### Ethical Considerations

This study was approved by the Ethics Committee of the Basic Medical College, Peking Union Medical College on March 10, 2021 (062‐2021). After expressing their initial interest, participants received ethically approved information about the purpose and procedures of this study. Those who chose to enroll indicated their willingness by submitting an online consent form. Data was gathered by KuRunData [24], ensuring participant anonymity from the research team, which only accessed deidentified data during the analysis phase. Throughout the trial and data analysis, the research team remained blinded to the specific assignments of individual participants. As the intervention involved video viewing, participants were aware of their own exposure. However, they were not informed of this study’s objectives or other experimental conditions, which helped reduce expectation bias. The anonymity of responses and restricted access to deidentified data only by the research team further minimized the risks of social desirability bias. Participants were rewarded with CN ¥5 (the 2021 official exchange rate of CN ¥1=US $0.155 was used for reference, based on World Bank data [25]) upon completing the survey.

### Study Design

We designed and implemented a nationwide single-blind, parallel-group, randomized controlled trial. The delivery of interventions and the collection of data were facilitated through computer webpages or smartphones. Participants in the intervention group submitted their sociodemographic details before viewing the animated video to which they were randomly assigned. Subsequently, we assessed their flu vaccine hesitancy using a validated survey tailored for our participant population. Participants in the control group were first asked to complete the survey before being granted access to the video interventions. This study was approved by the Ethics Committee of the Basic Medical College at Peking Union Medical College on March 10, 2021 (062‐2021). The trial was registered with the German Clinical Trials Register on February 9, 2021 (DRKS00023650).

### Participants

We targeted to recruit 12,000 Chinese adults aged 18 years or older, ensuring national representation through quota sampling based on age, gender, and residence (urban or rural) [26-28]. The quotas reflected the 2019 population estimates from the National Bureau of Statistics of China [29]. Additional details are available in Text S1 of Multimedia Appendix 1. Participants were sourced through KuRunData, a market research company with a membership base exceeding 17 million in China. Sampling was drawn from this large pool. Recruitment used various channels to achieve diverse and representative data. These channels included KuRunData’s proprietary software, digital advertisements, internet searches, word of mouth, member referrals, social media platforms such as TikTok (ByteDance), partner recommendations, and over 200 national coordinators who managed offline recruitment. This multisource approach significantly reduced reliance on any particular demographic or group.

While they were aware this was a health-related study, potential participants were not initially informed of the specific focus of our study.

### Randomization and Masking

Participants enrolled in this study were randomly allocated to 1 of 3 video intervention groups with different storytelling techniques (humor, analogy, and emotion) or a control group in equal proportions (1:1:1:1) using a computer-generated sequence that was independent of the researchers. Those in the intervention groups viewed 1 of the 3 intervention videos, without being informed of the other conditions. Each participant was assigned a unique, anonymous ID and required to complete a series of validated surveys to collect data.

### Enrollment Procedures

The recruitment phase of this study was conducted from April 15 to May 25, 2021. Interested participants were required to create an account on KuRunData. Upon agreeing to join our study, participants answered sociodemographic questions such as age, gender, residence type (urban or rural), education level, household annual income, and province. Intervention group participants then viewed their designated animated video intervention and completed the flu vaccine hesitancy measure (outcome measure) on either a computer or a smartphone. Control group participants filled out the questionnaire first and then were offered access to the intervention video. All procedures were completed in 1 session. To ensure the full video was played before proceeding to the next study task, the platform was programmed to prevent skipping. Moreover, prior research has shown that videos under 3 minutes in length fall within the optimal attention span and are associated with high viewer engagement and completion rates [30].

### Development of Intervention Videos

**Figure 1.**
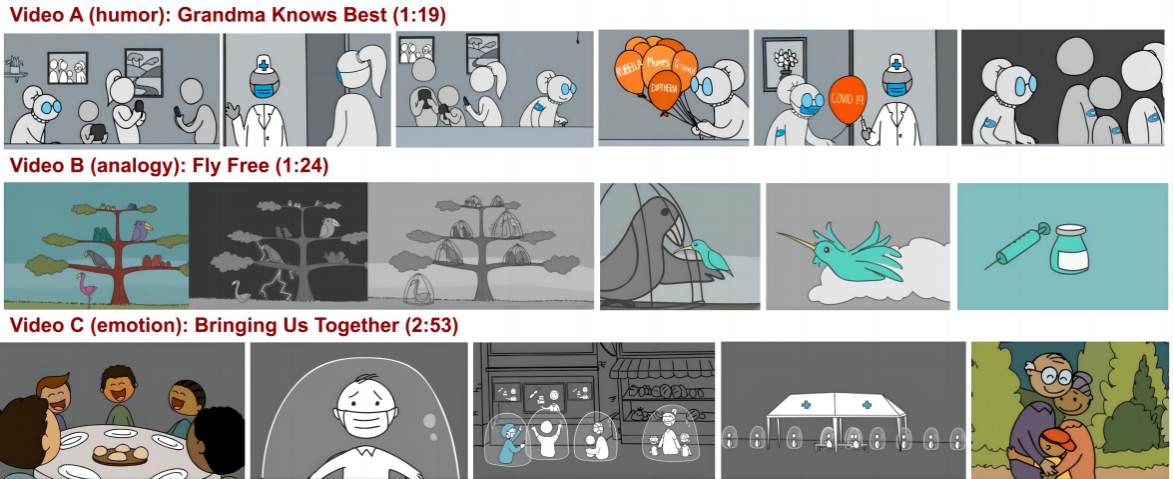
Screenshots from the video intervention.

We evaluated 3 short, animated entertainment-education videos, each ranging from 1 to 3 minutes in length. Video A, titled “grandma knows best,” used an instructional-humor strategy [31] featuring a quirky grandmother who is determined to get her family vaccinated. Video B, titled “fly free,” used a storytelling-analogy approach [32] in which caged birds symbolized people confined to their homes during the COVID-19 pandemic lockdowns. In this story, the birds only gained their freedom once they were vaccinated. Video C, titled “bringing us together,” focused on evoking emotion to connect with the audience [33]. This story portrayed the widespread feelings of loneliness and isolation experienced during the pandemic lockdowns, culminating in the joy of regaining individual freedoms after vaccination. While all videos featured narratives with the COVID-19 pandemic as the context, their main message was that vaccines are effective in preventing infections and promoting health. As such, they were designed to reduce vaccine hesitancy in general, including influenza vaccine hesitancy. Additionally, some psychological factors underlying vaccine hesitancy—such as fear of needles, concerns about safety and side effects, low perceived susceptibility to disease, and distrust in health authorities—are generalizable across different types of vaccines, providing a theoretical basis for the assumption of message generalizability and the design of the intervention [34-36]. Figure 1 displays selected screenshots from these interventions with each video using a unique instructional storytelling technique (ie, humor, analogy, and emotion).

The production of the intervention videos took place between November 2020 and March 2021 during the initial distribution of the first COVID-19 vaccines. We collaborated with an interdisciplinary team of experts, including those specializing in behavioral sciences, entertainment, and marketing. This approach allowed us to incorporate evidence-based vaccine-promotion messages into engaging and scalable short, animated entertainment-education videos. The character designs were culturally inclusive and based on insights from our previous study, which involved participants from 73 countries [37].

We used the principles of Universal Design for Learning to ensure the inclusiveness of the videos, with each of the 3 videos using slightly different methods [38]. Video A used iconic character representations without cultural identifiers and different shades of gray to indicate racial diversity. Video B illustrated diversity through various bird species. Video C adopted a hybrid approach, featuring an icon-style main character without cultural markers, with supporting characters representing diverse races and ethnicities. We also gathered formative input from stakeholders in China, Canada, South Africa, Germany, the United States, Mexico, and Australia. Our creative team, reflecting a global perspective, collaborated with international vaccine-promotion experts throughout the development process. Their feedback, shared via WhatsApp and Zoom, helped refine the video planning documents and drafts. This iterative and responsive process, recommended in human-centered design literature, ensured the interventions were continuously improved during production [39].

All 3 intervention videos were created in the same 2D animation style by the same animator. To maximize scalability across different language speakers, none of the videos included spoken language. This decision was influenced by the observed spontaneous distribution of our earlier wordless, animated entertainment-education content [20]. Instead of dialogue, each video relied on visual storytelling, complemented by an engaging soundtrack. The intervention videos can be accessed on YouTube via the following reference citation links: (1) video A (humor) [40], (2) video B (analogy) [41], and (3) video C (emotion) [42].

### Outcome

To assess participants’ hesitancy toward the influenza vaccine, we used the validated Adult Vaccine Hesitancy Scale (aVHS), which has been verified for the Chinese adult population [43,44]. Derived from the 14-item WHO Strategic Advisory Group of Experts on Immunization Vaccine Hesitancy Scale, the aVHS consists of 10 items, including 3 negative and 7 positive (reverse-coded) statements [45]. Participants rated their responses for each item on a 5-point scale, producing total scores ranging from 10 to 50, with higher scores indicating greater vaccine hesitancy.

### Statistical Analysis

Baseline characteristics included age, gender, residence, education level, household annual income, region, and economic belt, which were summarized by means (SDs) or n/N (%) as appropriate. To further differentiate the regions of China by their socioeconomic development status, we divided all regions into 4 economic belts, namely, East, Central, West, and Northeast [46]. We applied the Pearson *χ*^2^ test (with categorical variables) and Kruskal-Wallis rank sum test (with continuous variables) to compare baseline characteristics across groups. The primary analysis adhered to the intention-to-treat analysis principle. First, a pooled analysis combined participants from the 3 intervention groups and compared them with the control group using independent *t* tests for 2-tailed *P* values. Second, we evaluated flu vaccine hesitancy through 6 coprimary comparisons (each intervention group versus the control group, and comparisons between intervention groups). Bonferroni correction was applied to adjust for multiple pairwise comparisons [47]. The overall α level of .05 was evenly distributed across all coprimary comparisons; therefore, only *P* values (*P*_heterogeneity_) <.0083 were considered statistically significant [48]. For each coprimary comparison, we calculated the absolute difference in means and Cohen effect size *d* (Cohen *d*) along with nominal 99.17% CIs to maintain an overall α level of .05.

With a target of 5% type I error rate (2-sided) and a 20% type II error rate, a minimum sample size of 6280 was required to detect a 0.01 difference in mean vaccine hesitancy scores between groups (1=total acceptance, 5=complete refusal), assuming an SD of 0.10. We recruited 12,000 participants to account for multiple comparison adjustments. Given the extremely short duration of the interventions tested in this trial (1 to 3 minutes single exposures), enhanced statistical power was deemed necessary to detect any potential effects of these “micro-interventions” [15,49,50]. Detailed calculations are provided in this study’s protocol [16].

Subgroup analyses were conducted by baseline characteristics, with interaction *P* values calculated using likelihood ratio tests. Interaction *P* values (*P*_interaction_) <.05 were considered statistically significant. We differentiated between interaction effects, which examine significant differences in intervention effectiveness across subgroups, and heterogeneity, which indicates variability in effectiveness within subgroups [51]. All data and statistical analyses were performed in R (version 4.1.2; R Foundation).

## Results

### Sample Characteristics

**Figure 2.**
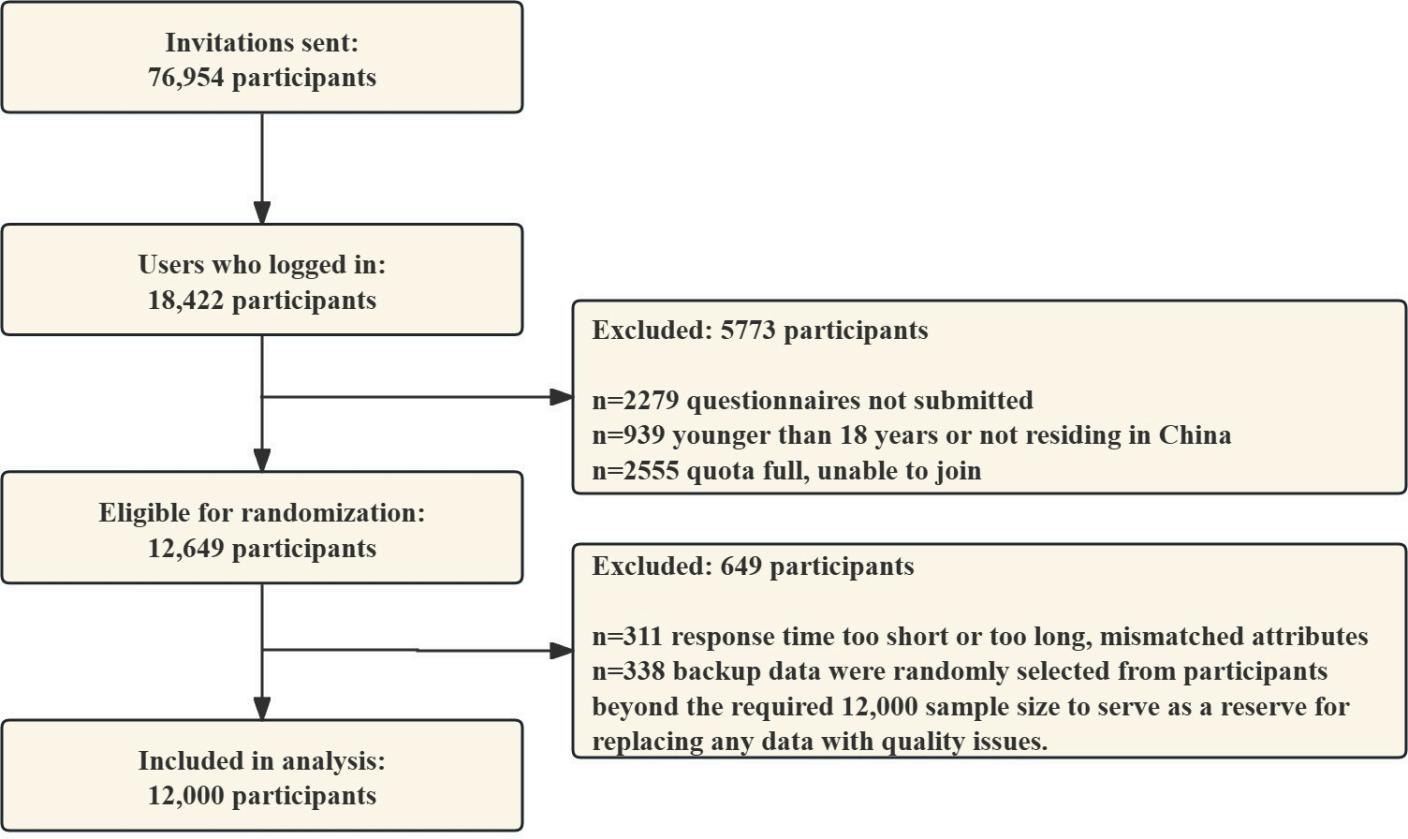
Study flowchart.

A total of 18,422 individuals indicated their interest in this study. Among them, 2279/18,422 (12.37%) were excluded for failing to submit the questionnaires, 939/18,422 (5.10%) were excluded for being younger than 18 years of age or not living in China, and 2555/18,422 (13.87%) were excluded due to reaching full participant quotas. As a result, 12,649/18,422 (68.66%) individuals met the initial eligibility criteria and were randomly assigned to intervention and control groups. Of these, 649/18,422 (3.52%) individuals were excluded because of insufficient response times or issues with backup data (randomly selected from participants beyond the required 12,000 to replace any low-quality data), leaving 12,000/18,422 (65.14%) participants included in the final analysis (Figure 2). In the final study cohort, the participants resided in 31 of the 32 provinces, autonomous regions, and municipalities of mainland China (Figure S1 in Multimedia Appendix 1). The average age of the participants was 44.3 years (SD 14.3), with 5873/12,000 (48.9%) women and 6127/12,000 (51.1%) men. Among them, 4744/12,000 (39.5%) lived in rural areas, 3600/12,000 (30%) had junior high or lower educational levels, 4411/12,000 (36.8%) had annual family incomes under CN ¥90,000, a total of 3680/12,000 (30.67%) were from the western region, and 4440/12,000 (37%) were from the western economic belt. The baseline characteristics were balanced across the intervention and control groups (Table 1). Additionally, data from the study period revealed that only 5 (0.04%) participants had a history of COVID-19.

**Table 1.** Baseline characteristics across randomization groups in the total study population.

Characteristic	Overall (n=12,000)	Control group (n=3000)	Video A (n=3000)	Video B (n=3000)	Video C (n=3000)	*P* value
Age (years), mean (SD)	44.3 (14.3)	44.2 (14.4)	44.2 (14.4)	44.1 (14.1)	44.7 (14.4)	.36
Age group (years), n (%)						.73
18‐29	2274 (19)	585 (19.5)	578 (19.3)	558 (18.6)	553 (18.4)	
30‐39	2355 (19.6)	575 (19.2)	578 (19.3)	621 (20.7)	581 (19.4)	
40‐49	2369 (19.7)	593 (19.8)	605 (20.2)	589 (19.6)	582 (19.4)	
50‐59	2290 (19.1)	568 (18.9)	561 (18.7)	590 (19.7)	571 (19)	
≥60	2712 (22.6)	679 (22.6)	678 (22.6)	642 (21.4)	713 (23.8)	
Sex, n (%)						.25
Male	6127 (51.1)	1504 (50.1)	1521 (50.7)	1578 (52.6)	1524 (50.8)	
Female	5873 (48.9)	1496 (49.9)	1479 (49.3)	1422 (47.4)	1476 (49.2)	
Residence, n (%)						.34
Rural	4744 (39.5)	1163 (38.8)	1211 (40.4)	1210 (40.3)	1160 (38.7)	
Urban	7256 (60.5)	1837 (61.2)	1789 (59.6)	1790 (59.7)	1840 (61.3)	
Education, n (%)						.25
Junior high school or below	3600 (30)	866 (28.9)	903 (30.1)	891 (29.7)	940 (31.3)	
High school or technical secondary school	3848 (32.1)	994 (33.1)	965 (32.2)	931 (31)	958 (31.9)	
College diploma or above	4552 (37.9)	1140 (38)	1132 (37.7)	1178 (39.3)	1102 (36.7)	
Total household income CN ¥^a^, n (%)						.83
Below 90,000	4411 (36.8)	1102 (36.7)	1118 (37.3)	1096 (36.5)	1095 (36.5)	
90,000 to 180,000	5172 (43.1)	1296 (43.2)	1306 (43.5)	1278 (42.6)	1292 (43.1)	
Above 180,000	2417 (20.1)	602 (20.1)	576 (19.2)	626 (20.9)	613 (20.4)	
Region, n (%)						.71
Northeast China	1120 (9.3)	272 (9.1)	312 (10.4)	270 (9)	266 (8.9)	
East China	2840 (23.7)	717 (23.9)	679 (22.6)	737 (24.6)	707 (23.6)	
North China	2000 (16.7)	504 (16.8)	507 (16.9)	478 (15.9)	511 (17)	
Central China	1200 (10)	313 (10.4)	299 (10)	299 (10)	289 (9.6)	
South China	1160 (9.7)	282 (9.4)	309 (10.3)	280 (9.3)	289 (9.6)	
Southwest China	1880 (15.7)	453 (15.1)	463 (15.4)	488 (16.3)	476 (15.9)	
Northwest China	1800 (15)	459 (15.3)	431 (14.4)	448 (14.9)	462 (15.4)	
Economic belt, n (%)						.73
East China	4080 (34)	1024 (34.1)	1022 (34.1)	1019 (34)	1015 (33.8)	
Central China	2360 (19.7)	586 (19.5)	583 (19.4)	595 (19.8)	596 (19.9)	
Northeast China	1120 (9.3)	272 (9.1)	312 (10.4)	270 (9)	266 (8.9)	
West China	4440 (37)	1118 (37.3)	1083 (36.1)	1116 (37.2)	1123 (37.4)	

a All monetary values are presented in Chinese yuan (CN ¥). The 2021 official exchange rate of CN ¥1=US $0.155 was used for reference, based on World Bank data [25].

### Overall Effectiveness of Short, Animated Storytelling Intervention

**Figure 3.**
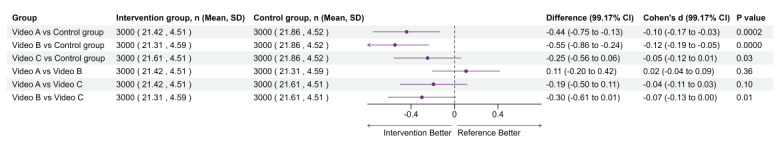
Effects of animated videos on flu vaccine hesitancy. We conducted 6 comparisons using a Bonferroni-adjusted α-level of .0083, presenting 99.17% CIs to maintain an overall α-level of .05, with *P* value <.0083 deemed statistically significant.

In the main analysis, participants in any of the 3 intervention groups showed lower flu vaccine hesitancy compared with the control group, with a mean difference of −0.41 (95% CI −0.60 to −0.23; *P* value <.001) measured in aVHS scores. Specifically, participants who received video A intervention showed significantly lower flu vaccine hesitancy than the control group (−0.44, 99.17% CI −0.75 to −0.13; *P* value =.0002); those who received intervention with video B also showed lower hesitancy compared to the control group (−0.55, 99.17% CI −0.86 to −0.24; *P* value <.001; Figure 3). However, no significant difference in the flu vaccination hesitancy was observed between the intervention groups with video A (humor) versus video B (analogy). Additionally, no significant effects were observed in the video C (emotion) group compared to the control group. Similarly, no significant differences were found when comparing video C (emotion) with other intervention groups with either video A (humor) or video B (analogy; Figure 3).

### Subgroup Analysis

**Figure 4.**
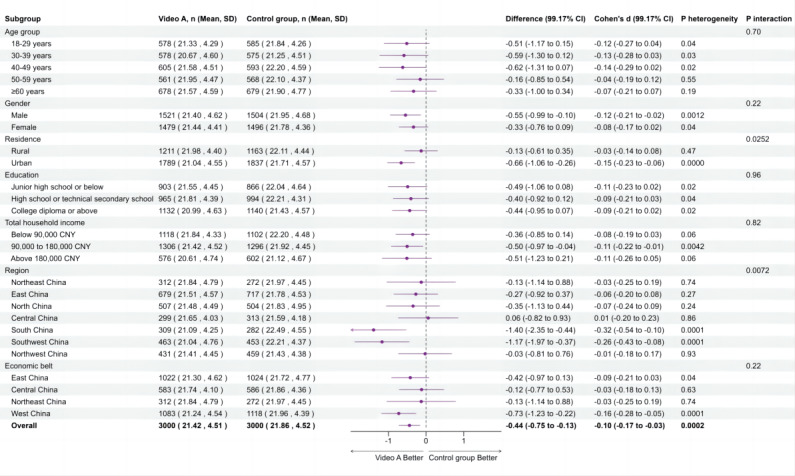
Effects of animated video A on flu vaccine hesitancy by subgroup. Interaction *P* values below .05 were considered statistically significant.

Subgroup analyses were performed for the comparisons between intervention group video A (humor) versus control group, video B (analogy) versus control group, and video C (emotion) versus control group, respectively. The forest plots illustrating the effectiveness of each animated video compared to the control group, moderated by factors such as age, gender, residence, education level, household annual income, region, and economic belt, are presented in Figures4^6^. In these subgroup analyses, a negative mean difference implies that the intervention group had lower hesitancy scores than the control group, meaning a positive effect of the intervention on reducing the flu vaccination hesitancy.

**Figure 5.**
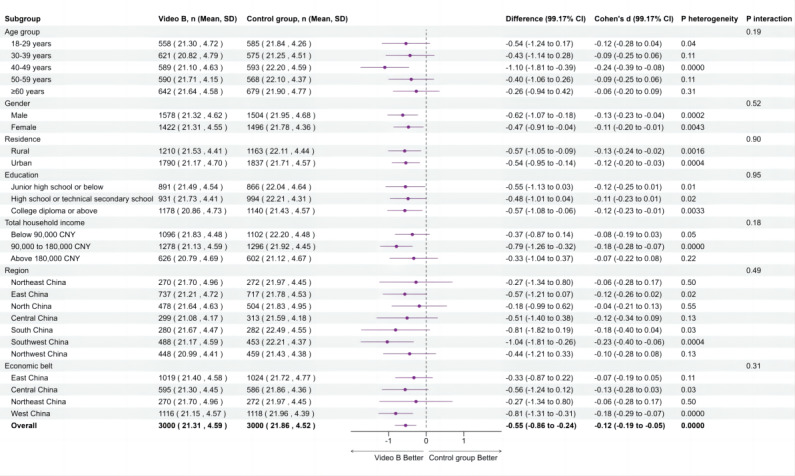
Effects of animated video B on flu vaccine hesitancy by subgroup. Interaction *P* values below .05 were considered statistically significant.

**Figure 6.**
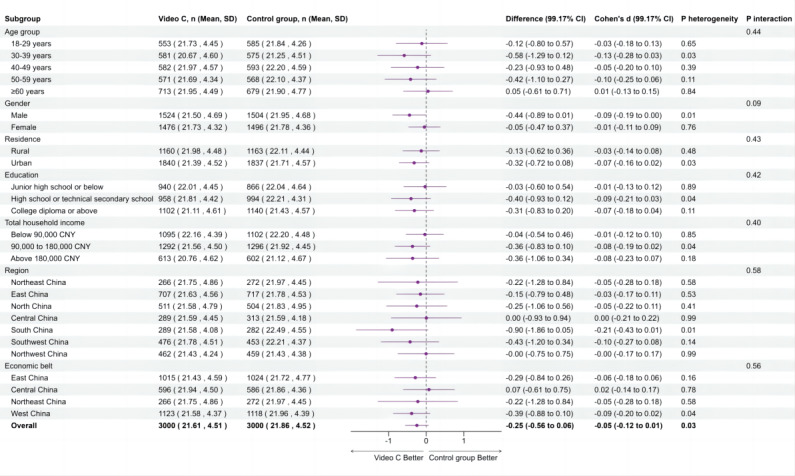
Effects of animated video C on flu vaccine hesitancy by subgroup. Interaction *P* values below .05 were deemed statistically significant.

In particular, the intervention group with video A (humor) showed significantly lower hesitancy compared to the control group when stratified by residence and region. Participants in urban areas (−0.66, 99.17% CI −1.06 to −0.26; *P*_interaction_=.0252), the South (−1.40, 99.17% CI −2.35 to −0.44), and the Southwest (−1.17, 99.17% CI −1.97 to −0.37; *P*_interaction_=.0072) exhibited greater reductions in flu vaccine hesitancy than the control group. Additionally, significant heterogeneity was observed within subgroups of males, urban residents, households with an income of CN ¥90,000‐180,000, the south and southwest region, and the western economic belt (*P*_heterogeneity_<.0083), indicating that video A reduced vaccine hesitancy within these subgroups. Although video B significantly reduced flu vaccine hesitancy overall compared with the control group, the interaction effects between subgroups were not significant (*P*_interaction_>.05), indicating no significant differences in intervention effects among different subgroups. However, significant heterogeneity was observed within certain subgroups, including those aged 40‐49 years, males, females, rural residents, urban residents, participants with a college education or higher, households with an income of CN ¥90,000‐180,000, the southwest region, and the western economic belt (*P*_heterogeneity_<.0083), indicating that video B reduced vaccine hesitancy within these subgroups. Video C did not show a significant overall effect or differences by subgroup.

## Discussion

### Principal Findings

This large-scale, nationwide, single-blind, parallel-group randomized controlled trial demonstrated the effectiveness of short, animated storytelling videos, using humor and analogy as storytelling techniques, for reducing influenza vaccine hesitancy among a diverse adult population in China. This specialized approach to entertainment-education significantly reduced vaccine hesitancy, highlighting the potential of culturally accessible, wordless short, animated storytelling videos to support scalable public health messaging. Furthermore, the observed decrease in influenza vaccine hesitancy achieved through short, animated storytelling narratives built in the context of COVID-19 underscores the potential synergies of provaccination messages to boost vaccine uptake across different vaccines.

The main results in this study add to an emerging body of literature that is focused on the study of the short, animated storytelling approach to public health entertainment-education [15,17,52,53]. According to health behavior change theories, fostering positive attitudes toward a behavior significantly enhances an individual’s intention to engage in that behavior [54]. This foundational theory supports the observed effectiveness of the short, animated storytelling approach in this study. Additionally, this study suggests that humor and analogies might be more effective than emotional appeals in reducing vaccine hesitancy. Humor can relieve stress and transform negative emotions into positive emotions, helping to engage audiences more effectively and potentially making the educational content more persuasive [55]. However, analogies simplify complex information, making it more comprehensible across diverse demographics, including different ages, genders, and educational levels [56,57]. In contrast, emotional messages can sometimes be complex and open to interpretation, which may reduce their clarity and effectiveness. Additionally, during the COVID-19 pandemic, the public was exposed to a high volume of health messages, many of which used emotional appeals. This information overload may have led to diminishing returns of repeated emotional messaging among individuals [58]. While prior interventions aimed at improving vaccine literacy and building trust have been effective in shifting attitudes, such efforts have traditionally been text-heavy or linguistically specific, limiting their reach in linguistically diverse populations [59,60]. Our study contributes to this understanding by demonstrating that short, animated storytelling videos can significantly reduce vaccine hesitancy by addressing these concerns through engaging, wordless narratives.

Our findings on the effectiveness of short, animated storytelling, specifically using humor and analogy as storytelling techniques, for reducing vaccine hesitancy are aligned with the findings of prior studies in the extant literature. A systematic review on behavior change theory-based social media interventions demonstrated the potential of short-form interventions, guided by behavior change theories, for promoting measurable health behavior change [61]. Additionally, other studies have shown the effectiveness of using similar approaches of entertainment-education videos on reducing barriers to cervical cancer screening among Thai women [62], strengthening substance use prevention among 7th-grade students in the United States [63], and increasing intentions of coping with loneliness among participants in Germany [64]. Furthermore, results from a randomized controlled trial conducted among older adults aged older than 60 years in 8 communities of Xi’an, China found that video-led educational interventions significantly improved older adults’ willingness to get vaccinated, boosting influenza vaccine uptake [21]. However, findings from a randomized controlled trial evaluating the effectiveness of animated video- versus text-based educational interventions on influenza vaccine uptake among patients with inflammatory bowel diseases showed no significant difference between the 2 interventions, implying that other interventions may be needed to change long-term beliefs in this subpopulation [22]. Findings from another randomized controlled trial investigating the impact of short, animated storytelling videos on knowledge, behavioral intent, and engagement regarding COVID-19 vaccination showed significant knowledge gains and high engagement, especially among younger audiences, but only indirect effects on behavioral intent toward vaccination [18]. Considered alongside our findings, this body of evidence suggests both the potential for short, animated storytelling interventions to support public health initiatives, as well as the need for more research into this approach.

We also observed substantial disparities in the effectiveness of short, animated storytelling videos with humor and analogy by socioeconomic subgroups. For example, we found that the use of analogy in video B only showed significant reduction in vaccine hesitancy among highly educated individuals. This finding suggests that the use of analogy may be particularly effective among those with higher education levels, who tend to have greater awareness and understanding of health information, making them more receptive to the use of analogy in vaccine communication [65,66]. This enhanced awareness likely allows for more educated populations to appreciate the nuances and engage more deeply with the content, potentially yielding a greater impact on their attitudes and behaviors toward vaccination. Additionally, using humor and analogy in videos A and B, respectively, reduced vaccine hesitancy among people with higher income levels, potentially because these individuals often have more time and financial resources to consider health care decisions comprehensively, including the benefits and risks associated with vaccines [67]. Further, the observed effects of short, animated storytelling videos on vaccine hesitancy may be influenced by the heightened awareness around vaccines during the COVID-19 pandemic. It is possible that participants’ increased familiarity with vaccine-related health information contributed to the intervention’s effectiveness, and the intervention effectiveness may have varied across different socioeconomic subgroups, as populations with high income and education levels may have been more engaged with health information during COVID-19 [68]. Future research should evaluate the sustainability of these effects as pandemic-related health communication normalizes, particularly in regions where health care access expanded postpandemic. Conversely, tailored communication strategies may be needed to enhance vaccine uptake in low-income subgroups by addressing their specific needs.

Besides socioeconomic differences, our findings also demonstrate significant geographical differences in the effectiveness of the short, animated storytelling video interventions on reducing influenza vaccine hesitancy. Urban residents showed a greater reduction in vaccine hesitancy, particularly for those exposed to video A (humor). This may be because urban residents are frequently exposed to digital media, and this regular engagement with digital content might enhance the receptivity to humor-based health messages [69,70]. In fact, social media was found to be the most popular avenue to obtain influenza-related information in populations that have access to it [71]. Additionally, the effectiveness of videos A and B in the South (for video A only) and the Southwest may be due to the relatively higher income-level population groups residing in those regions. As discussed above, the analogy and humor in the videos may be more relatable and impactful in these higher-income groups, thereby addressing vaccine hesitancy more effectively. Furthermore, the difference in the intervention effect magnitude between urban and rural regions may stem partly from logistical barriers such as vaccination service availability. In regions where there is a lack of vaccination services, measured changes in vaccine hesitancy may reflect access issues rather than changes in attitude and intent [72,73]. Moreover, it is important to recognize that the full impact of short, animated storytelling videos on actual vaccination rates may only be realized if vaccination service accessibility is addressed concurrently.

The findings of this study have significant implications for public health policy and practice. The demonstrated effectiveness of using humor and analogies to reduce vaccine hesitancy could inform public health strategies by highlighting the importance of engaging, relatable, and culturally sensitive communication methods. Policy makers may integrate these findings into vaccination campaigns to enhance public receptivity, engagement, and compliance. Regarding scalability, the intervention shows promise for broader application across larger populations and different health issues. By using digital media, the approach can reach diverse audiences efficiently, making it a viable option for nationwide health campaigns. Furthermore, the customization potential of this strategy is considerable. It can be adapted to suit various demographic groups and regions with distinct cultural contexts by tailoring the humor and analogies to reflect local languages, customs, and health challenges.

This study boasts several notable strengths, beginning with its robust design. The use of a randomized controlled trial ensures high internal validity, allowing for straightforward causal findings about the intervention’s effects. Additionally, the large sample size enhances the statistical power and reliability of the findings by including participants from various regions and demographic groups across China, while the use of a validated vaccine hesitancy scale ensures that the measures are accurate and meaningful. However, our study has several limitations. The reliance on self-reported data introduces potential biases, as participants may underreport or overreport their vaccine hesitancy due to social desirability or recall biases. Conducting the intervention and data collection online poses additional limitations, including issues of digital access and engagement. Participants without reliable internet access or those less comfortable with digital platforms may be underrepresented, potentially skewing the results. Moreover, the differences noted within subgroups should be interpreted with caution due to the small sample sizes in some cases. Lastly, the generalizability of these findings beyond the setting of China may be limited. While this study includes a diverse sample, the cultural and regional differences within China and between other countries could affect the applicability of the results to other populations or settings. These limitations suggest the need for further research to validate the findings across different contexts.

### Conclusions

This study is the first large-scale, nationwide, randomized controlled trial to evaluate the effectiveness of short, animated storytelling videos in reducing influenza vaccine hesitancy among Chinese adults. Our findings demonstrate that short, animated storytelling videos using humor and analogy as storytelling techniques significantly decreased vaccine hesitancy compared to the control group. These results underscore the potential of short, animated storytelling videos as a scalable, low-cost intervention to address vaccine hesitancy, providing policy makers with actionable insights with regards to increasing influenza vaccination rates in China. Policy makers should consider integrating this strategy into broader immunization campaigns to enhance vaccine literacy, facilitate effective health communication, and ultimately improve vaccination coverage. It would be beneficial to follow up with participants in this study to assess the duration of the intervention’s impact as a next step. Future research should also explore the long-term impact of such interventions and their applicability to other health behaviors and global settings.

## Supplementary material

10.2196/66758 Multimedia Appendix 1 Quota sampling procedure and geographic distribution of participants across China.

10.2196/66758 Checklist 1 CONSORT-eHEALTH checklist (V 1.6.1). CONSORT: Consolidated Standards of Reporting Trials.

## Abbreviations

aVHS: Adult Vaccine Hesitancy Scale
WHO: World Health Organization
