# Supplementary material for: Effects of Scalable, Wordless, Short, Animated Storytelling Videos on Flu Vaccine Hesitancy in China: Nationwide, Single-Blind, Parallel-Group, Randomized Controlled Trial
Source: J Med Internet Res. 2025 Aug 27;27:e66758. doi: 10.2196/66758 (PMC12385612; doi:10.2196/66758)
Supplement: Multimedia Appendix 1 — Quota sampling procedure and geographic distribution of participants across China. [file jmir-v27-e66758-s001.docx]

[Text S1. Quota sampling process. 2](#_Toc203233839)

[Figure S1. Nationwide reach of participants in China. 3](#_Toc203233840)

# Text S1. Quota sampling process.

1. Across China’s 31 provinces, autonomous regions, and municipalities, each was initially allocated an average quota of 360 participants.

2. For regions with populations exceeding 40 million, the quota was increased to 400 participants.

3. Quotas were distributed by urban/rural status, gender, and age, based on the 2019 population estimates from the National Bureau of Statistics of China. In Beijing, Shanghai, and Tianjin, rural male and female quotas were below 50; adjustments were made as follows:

-Step 1: Increase the rural male and female quotas in Beijing, Shanghai, and Tianjin to 50 participants each.

-Step 2: To account for the increase in rural quotas, the quotas for Beijing and Shanghai were raised from 400 to 480 participants each, while Tianjin's quota was adjusted from 360 to 400 participants, resulting in a total sample size of 12,000.

-Step 3: Determine the urban quotas for Beijing, Shanghai, and Tianjin by deducting the rural quotas from each city's overall quota. Subsequently, allocate these urban quotas between males and females based on the gender ratio.

# Figure S1. Nationwide reach of participants in China.


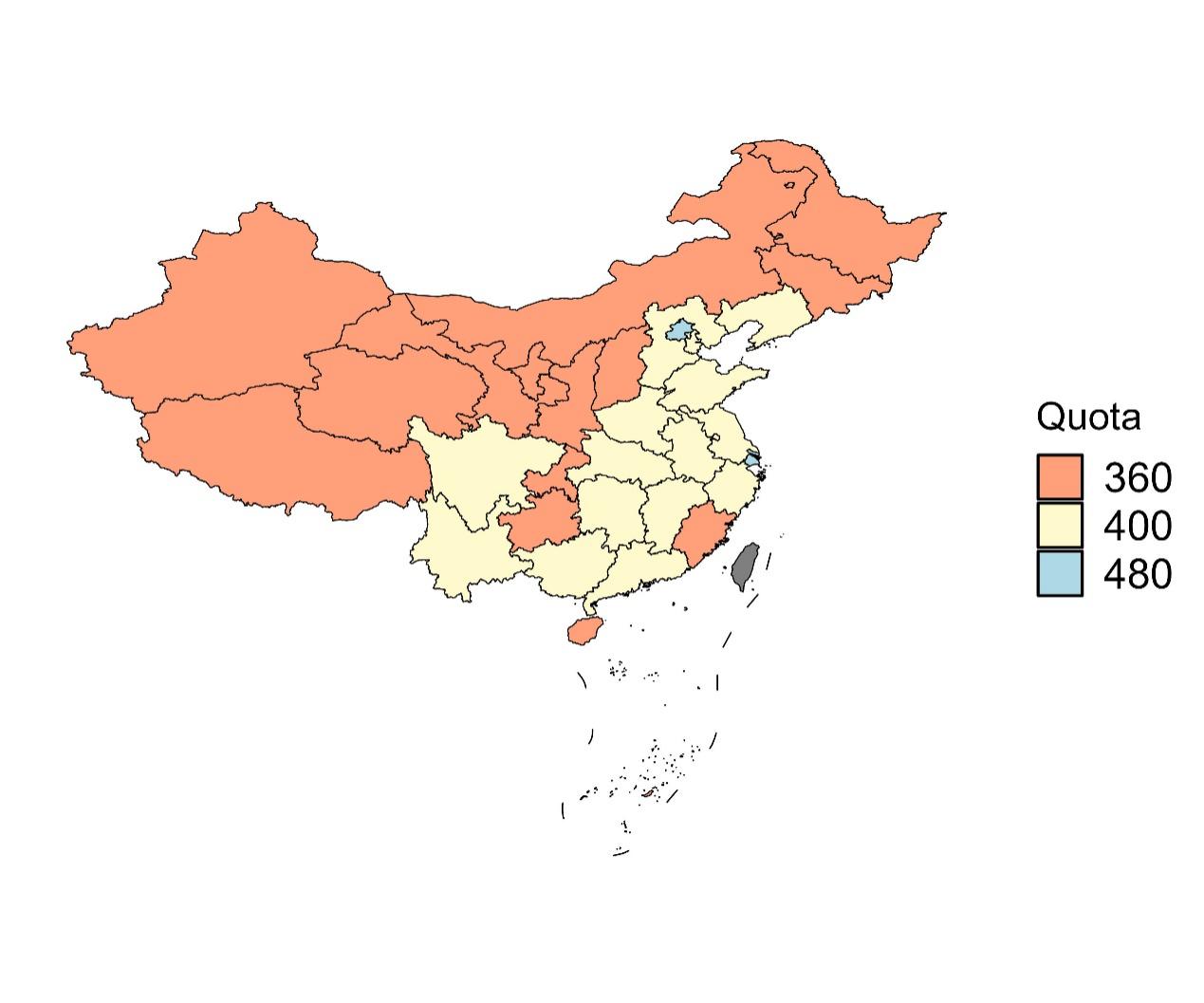


Shanxi, Inner Mongolia, Jilin, Heilongjiang, Fujian, Hainan, Chongqing, Guizhou, Tibet, Shaanxi, Gansu, Qinghai, Ningxia, and Xinjiang each have a quota of 360 participants. Provinces with populations exceeding 40 million, such as Hebei, Liaoning, Jiangsu, Zhejiang, Anhui, Jiangxi, Shandong, Henan, Hubei, Hunan, Guangdong, Guangxi, Sichuan, and Yunnan, have been allocated a higher quota of 400 participants. In addition, due to their higher urban population densities, the quotas for Beijing and Shanghai have been set at 480, while Tianjin's quota has been adjusted to 400.
