# Supplementary material for: Effects of Scalable, Wordless, Short, Animated Storytelling Videos on Flu Vaccine Hesitancy in China: Nationwide, Single-Blind, Parallel-Group, Randomized Controlled Trial
Source: J Med Internet Res. 2025 Aug 27;27:e66758. doi: 10.2196/66758 (PMC12385612; doi:10.2196/66758)
Supplement: Checklist 1 — CONSORT-eHEALTH checklist (V 1.6.1). CONSORT: Consolidated Standards of Reporting Trials. [file jmir-v27-e66758-s002.pdf]

CONSORT-EHEALTH (V 1.6.1) - Submission/Publication Form

The CONSORT-EHEALTH checklist is intended for authors of randomized trials evaluating web-based and Internet-based applications/interventions, including mobile interventions, electronic games (incl multiplayer games), social media, certain telehealth applications, and other interactive and/or networked electronic applications. Some of the items (e.g. all subitems under item 5 - description of the intervention) may also be applicable for other study designs.

The goal of the CONSORT EHEALTH checklist and guideline is to be
a) a guide for reporting for authors of RCTs,
b) to form a basis for appraisal of an ehealth trial (in terms of validity)

CONSORT-EHEALTH items/subitems are MANDATORY reporting items for studies published in the Journal of Medical Internet Research and other journals / scientific societies endorsing the checklist.

Items numbered 1., 2., 3., 4a., 4b etc are original CONSORT or CONSORT-NPT (non-pharmacologic treatment) items.
Items with Roman numerals (i., ii, iii, iv etc.) are CONSORT-EHEALTH extensions/clarifications.

As the CONSORT-EHEALTH checklist is still considered in a formative stage, we would ask that you also RATE ON A SCALE OF 1-5 how important/useful you feel each item is FOR THE PURPOSE OF THE CHECKLIST and reporting guideline (optional).

Mandatory reporting items are marked with a red *.
In the textboxes, either copy & paste the relevant sections from your manuscript into this form - please include any quotes from your manuscript in QUOTATION MARKS,
or answer directly by providing additional information not in the manuscript, or elaborating on why the item was not relevant for this study.

YOUR ANSWERS WILL BE PUBLISHED AS A SUPPLEMENTARY FILE TO YOUR PUBLICATION IN JMIR AND ARE CONSIDERED PART OF YOUR PUBLICATION (IF ACCEPTED).
Please fill in these questions diligently. Information will not be copyedited, so please use proper spelling and grammar, use correct capitalization, and avoid abbreviations.

DO NOT FORGET TO SAVE AS PDF _AND_ CLICK THE SUBMIT BUTTON SO YOUR ANSWERS ARE IN OUR DATABASE !!!

Citation Suggestion (if you append the pdf as Appendix we suggest to cite this paper in the caption):
Eysenbach G, CONSORT-EHEALTH Group
CONSORT-EHEALTH: Improving and Standardizing Evaluation Reports of Web-based and Mobile Health Interventions
J Med Internet Res 2011;13(4):e126

URL: http://www.jmir.org/2011/4/e126/
doi: 10.2196/jmir.1923 IF: 5.8 Q1 PMID: 22209829 IF: 5.8 Q1

feierli77@gmail.com 切换账号
未共享的内容

草稿已保存

* 表示必填

Your name *

First Last

Wenjin Chen

Primary Affiliation (short), City, Country *

University of Toronto, Toronto, Canada

PUMC, Beijing, China

Your e-mail address *

abc@gmail.com

wenjinchen2020@163.com

Title of your manuscript *

Provide the (draft) title of your manuscript.

Effects of scalable, wordless, short, animated storytelling videos on flu vaccine hesitancy in China: a nationwide, single-blind, parallel-group, randomized controlled trial

Name of your App/Software/Intervention *

If there is a short and a long/alternate name, write the short name first and add the long name in brackets.

Video A, titled "Grandma Knows Best"; Video B

Evaluated Version (if any)

e.g. "V1", "Release 2017-03-01", "Version 2.0.27913"

您的回答

Language(s) *

What language is the intervention/app in? If multiple languages are available, separate by comma (e.g. "English, French")

wordless

URL of your Intervention Website or App

e.g. a direct link to the mobile app on app in appstore (itunes, Google Play), or URL of the website. If the intervention is a DVD or hardware, you can also link to an Amazon page.

https://youtu.be/ap8xpyREaTc https://youtu.be/fYYBJ0d6gl0 https://youtu.be/WH5KUhGtfa[illegible]

URL of an image/screenshot (optional)

您的回答

Accessibility *

Can an enduser access the intervention presently?

- (selected) access is free and open
- access only for special usergroups, not open
- access is open to everyone, but requires payment/subscription/in-app purchases
- app/intervention no longer accessible
- 其他:

Primary Medical Indication/Disease/Condition *

e.g. "Stress", "Diabetes", or define the target group in brackets after the condition, e.g. "Autism (Parents of children with)", "Alzheimers (Informal Caregivers of)"

vaccine hesitancy

Primary Outcomes measured in trial *

comma-separated list of primary outcomes reported in the trial

vaccine hesitancy

Secondary/other outcomes

Are there any other outcomes the intervention is expected to affect?

vaccination intention; hope

Recommended "Dose" *

What do the instructions for users say on how often the app should be used?

- ○ Approximately Daily
- ○ Approximately Weekly
- ○ Approximately Monthly
- ○ Approximately Yearly
- ◉ "as needed"
- ○ 其他: ____________________

Approx. Percentage of Users (starters) still using the app as recommended after 3 months *

- ◉ unknown / not evaluated
- ○ 0-10%
- ○ 11-20%
- ○ 21-30%
- ○ 31-40%
- ○ 41-50%
- ○ 51-60%
- ○ 61-70%
- ○ 71%-80%
- ○ 81-90%
- ○ 91-100%
- ○ 其他: ____________________

Overall, was the app/intervention effective? *

- (selected) yes: all primary outcomes were significantly better in intervention group vs control
- partly: SOME primary outcomes were significantly better in intervention group vs control
- no statistically significant difference between control and intervention
- potentially harmful: control was significantly better than intervention in one or more outcomes
- inconclusive: more research is needed
- 其他: ______

Article Preparation Status/Stage *

At which stage in your article preparation are you currently (at the time you fill in this form)

- not submitted yet - in early draft status
- not submitted yet - in late draft status, just before submission
- (selected) submitted to a journal but not reviewed yet
- submitted to a journal and after receiving initial reviewer comments
- submitted to a journal and accepted, but not published yet
- published
- 其他: ______

Journal *

If you already know where you will submit this paper (or if it is already submitted), please provide the journal name (if it is not JMIR, provide the journal name under "other")

- ( ) not submitted yet / unclear where I will submit this
- (•) Journal of Medical Internet Research (JMIR)
- ( ) JMIR mHealth and UHealth
- ( ) JMIR Serious Games
- ( ) JMIR Mental Health
- ( ) JMIR Public Health
- ( ) JMIR Formative Research
- ( ) Other JMIR sister journal
- ( ) 其他:

Is this a full powered effectiveness trial or a pilot/feasibility trial? *

- ( ) Pilot/feasibility
- (•) Fully powered

Manuscript tracking number *

If this is a JMIR submission, please provide the manuscript tracking number under "other" (The ms tracking number can be found in the submission acknowledgement email, or when you login as author in JMIR. If the paper is already published in JMIR, then the ms tracking number is the four-digit number at the end of the DOI, to be found at the bottom of each published article in JMIR)

- ( ) no ms number (yet) / not (yet) submitted to / published in JMIR
- (•) 其他: Manuscript ID: 66758

TITLE AND ABSTRACT

1a) TITLE: Identification as a randomized trial in the title

1a) Does your paper address CONSORT item 1a? *

I.e does the title contain the phrase "Randomized Controlled Trial"? (if not, explain the reason under "other")

- (selected) yes
- 其他: ____

1a-i) Identify the mode of delivery in the title

Identify the mode of delivery. Preferably use “web-based” and/or “mobile” and/or “electronic game” in the title. Avoid ambiguous terms like “online”, “virtual”, “interactive”. Use “Internet-based” only if Intervention includes non-web-based Internet components (e.g. email), use “computer-based” or “electronic” only if offline products are used. Use “virtual” only in the context of “virtual reality” (3-D worlds). Use “online” only in the context of “online support groups”. Complement or substitute product names with broader terms for the class of products (such as “mobile” or “smart phone” instead of “iphone”), especially if the application runs on different platforms.

	1	2	3	4	5	
subitem not at all important	○	○	○	○	○	essential

Does your paper address subitem 1a-i? *

Copy and paste relevant sections from manuscript title (include quotes in quotation marks "like this" to indicate direct quotes from your manuscript), or elaborate on this item by providing additional information not in the ms, or briefly explain why the item is not applicable/relevant for your study

Explanation of Non-Applicability for Subitem 1a-i: Although our study title, "Effects of scalable, wordless, short, animated storytelling videos on flu vaccine hesitancy in China: a nationwide, single-blind, parallel-group, randomized controlled trial," does not directly employ terms such as “web-based” or “mobile,” our videos are disseminated through online platforms like YouTube. The core of our research is to evaluate how these animated videos impact audiences via online platforms, not the specifics of the delivery technology or method itself. Therefore, while the videos are indeed delivered through an internet platform, our title focuses on the medical outcomes of the study rather than the technical details of implementation. Thus, subitem 1a-i is not directly relevant to our research as it primarily concentrates on the delivery mode, which, while implicit in our method of video dissemination, is not the primary focus of our title or research inquiry.

1a-ii) Non-web-based components or important co-interventions in title

Mention non-web-based components or important co-interventions in title, if any (e.g., “with telephone support”).

	1	2	3	4	5	
subitem not at all important	○	○	○	○	○	essential

Does your paper address subitem 1a-ii?

Copy and paste relevant sections from manuscript title (include quotes in quotation marks "like this" to indicate direct quotes from your manuscript), or elaborate on this item by providing additional information not in the ms, or briefly explain why the item is not applicable/relevant for your study

您的回答

1a-iii) Primary condition or target group in the title

Mention primary condition or target group in the title, if any (e.g., “for children with Type I Diabetes”) Example: A Web-based and Mobile Intervention with Telephone Support for Children with Type I Diabetes: Randomized Controlled Trial

	1	2	3	4	5	
subitem not at all important	○	○	○	○	○	essential

Does your paper address subitem 1a-iii? *

Copy and paste relevant sections from manuscript title (include quotes in quotation marks "like this" to indicate direct quotes from your manuscript), or elaborate on this item by providing additional information not in the ms, or briefly explain why the item is not applicable/relevant for your study

Effects of scalable, wordless, short, animated storytelling videos on "flu vaccine hesitancy" in China: a nationwide, single-blind, parallel-group, randomized controlled trial

1b) ABSTRACT: Structured summary of trial design, methods, results, and conclusions

NPT extension: Description of experimental treatment, comparator, care providers, centers, and blinding status.

1b-i) Key features/functionalities/components of the intervention and comparator in the METHODS section of the ABSTRACT

Mention key features/functionalities/components of the intervention and comparator in the abstract. If possible, also mention theories and principles used for designing the site. Keep in mind the needs of systematic reviewers and indexers by including important synonyms. (Note: Only report in the abstract what the main paper is reporting. If this information is missing from the main body of text, consider adding it)

	1	2	3	4	5	
subitem not at all important	○	○	○	○	○	essential

Does your paper address subitem 1b-i? *

Copy and paste relevant sections from the manuscript abstract (include quotes in quotation marks "like this" to indicate direct quotes from your manuscript), or elaborate on this item by providing additional information not in the ms, or briefly explain why the item is not applicable/relevant for your study

"In this single-blind, parallel-group, randomized controlled trial, we recruited adults in China through quota sampling. Participants were randomly assigned to one of three SAS video intervention groups, each using a different storytelling technique (humor, analogy, or emotion) or a control group in a 1:1:1:1 ratio. "

1b-ii) Level of human involvement in the METHODS section of the ABSTRACT

Clarify the level of human involvement in the abstract, e.g., use phrases like “fully automated” vs. “therapist/nurse/care provider/physician-assisted” (mention number and expertise of providers involved, if any). (Note: Only report in the abstract what the main paper is reporting. If this information is missing from the main body of text, consider adding it)

	1	2	3	4	5	
subitem not at all important	○	○	○	○	○	essential

Does your paper address subitem 1b-ii?

Copy and paste relevant sections from the manuscript abstract (include quotes in quotation marks "like this" to indicate direct quotes from your manuscript), or elaborate on this item by providing additional information not in the ms, or briefly explain why the item is not applicable/relevant for your study

您的回答

1b-iii) Open vs. closed, web-based (self-assessment) vs. face-to-face assessments in the METHODS section of the ABSTRACT

Mention how participants were recruited (online vs. offline), e.g., from an open access website or from a clinic or a closed online user group (closed usergroup trial), and clarify if this was a purely web-based trial, or there were face-to-face components (as part of the intervention or for assessment). Clearly say if outcomes were self-assessed through questionnaires (as common in web-based trials). Note: In traditional offline trials, an open trial (open-label trial) is a type of clinical trial in which both the researchers and participants know which treatment is being administered. To avoid confusion, use “blinded” or “unblinded” to indicated the level of blinding instead of “open”, as “open” in web-based trials usually refers to “open access” (i.e. participants can self-enrol). (Note: Only report in the abstract what the main paper is reporting. If this information is missing from the main body of text, consider adding it)

	1	2	3	4	5	
subitem not at all important	○	○	○	○	○	essential

Does your paper address subitem 1b-iii?

Copy and paste relevant sections from the manuscript abstract (include quotes in quotation marks "like this" to indicate direct quotes from your manuscript), or elaborate on this item by providing additional information not in the ms, or briefly explain why the item is not applicable/relevant for your study

您的回答

1b-iv) RESULTS section in abstract must contain use data

Report number of participants enrolled/assessed in each group, the use/uptake of the intervention (e.g., attrition/adherence metrics, use over time, number of logins etc.), in addition to primary/secondary outcomes. (Note: Only report in the abstract what the main paper is reporting. If this information is missing from the main body of text, consider adding it)

	1	2	3	4	5	
subitem not at all important	○	○	○	○	○	essential

Does your paper address subitem 1b-iv?

Copy and paste relevant sections from the manuscript abstract (include quotes in quotation marks "like this" to indicate direct quotes from your manuscript), or elaborate on this item by providing additional information not in the ms, or briefly explain why the item is not applicable/relevant for your study

您的回答

1b-v) CONCLUSIONS/DISCUSSION in abstract for negative trials

Conclusions/Discussions in abstract for negative trials: Discuss the primary outcome - if the trial is negative (primary outcome not changed), and the intervention was not used, discuss whether negative results are attributable to lack of uptake and discuss reasons. (Note: Only report in the abstract what the main paper is reporting. If this information is missing from the main body of text, consider adding it)

	1	2	3	4	5	
subitem not at all important	○	○	○	○	○	essential

Does your paper address subitem 1b-v?

Copy and paste relevant sections from the manuscript abstract (include quotes in quotation marks "like this" to indicate direct quotes from your manuscript), or elaborate on this item by providing additional information not in the ms, or briefly explain why the item is not applicable/relevant for your study

您的回答

INTRODUCTION

2a) In INTRODUCTION: Scientific background and explanation of rationale

2a-i) Problem and the type of system/solution

Describe the problem and the type of system/solution that is object of the study: intended as stand-alone intervention vs. incorporated in broader health care program? Intended for a particular patient population? Goals of the intervention, e.g., being more cost-effective to other interventions, replace or complement other solutions? (Note: Details about the intervention are provided in “Methods” under 5)

	1	2	3	4	5	
subitem not at all important	○	○	○	○	○	essential

Does your paper address subitem 2a-i? *

Copy and paste relevant sections from the manuscript (include quotes in quotation marks "like this" to indicate direct quotes from your manuscript), or elaborate on this item by providing additional information not in the ms, or briefly explain why the item is not applicable/relevant for your study

"Influenza is a highly contagious respiratory illness that poses a significant threat to human health. Approximately one billion cases of seasonal influenza are documented each year, including 3 to 5 million severe cases.1 Caused by the influenza virus, “the flu” results in 290,000 to 650,000 deaths each year worldwide.1 In low- and middle-income countries (LMICs), the risk of severe influenza cases is higher than in high-income countries.2 In 2021, more than 668,200 influenza cases, contributing to 10.72% of the total global cases, occurred in China.3

Influenza vaccination is highly effective in preventing influenza and reducing morbidity and mortality associated with this disease.4 Studies have documented the cost-effectiveness of influenza vaccination for improving influenza outcomes.5,6 Yet vaccine hesitancy, defined by the World Health Organization (WHO) as the "delay in acceptance or refusal of vaccines despite the availability of vaccination services," presents a major obstacle to achieving adequate vaccination coverage and the WHO has identified vaccine hesitancy as one of the top ten threats to global health.7"

2a-ii) Scientific background, rationale: What is known about the (type of) system

Scientific background, rationale: What is known about the (type of) system that is the object of the study (be sure to discuss the use of similar systems for other conditions/diagnoses, if appropiate), motivation for the study, i.e. what are the reasons for and what is the context for this specific study, from which stakeholder viewpoint is the study performed, potential impact of findings [2]. Briefly justify the choice of the comparator.

	1	2	3	4	5	
subitem not at all important	○	○	○	○	○	essential

Does your paper address subitem 2a-ii? *

Copy and paste relevant sections from the manuscript (include quotes in quotation marks "like this" to indicate direct quotes from your manuscript), or elaborate on this item by providing additional information not in the ms, or briefly explain why the item is not applicable/relevant for your study

"In China, influenza vaccination rates remain low, with a recent global meta-analysis reporting flu vaccine coverage of less than 17% in the general population.8 Furthermore, despite Chinese CDC's vaccine recommendations for high risk populations, studies found that vaccination rates were even lower (14.12%) for those with chronic diseases.8 Even among healthcare workers in China, less than one in four are protected by influenza vaccination (23.1% as reported in a recent global meta-analysis).8 In China, important contributors to vaccine hesitancy include concerns about the side effects, safety and efficacy of influenza vaccines.9-11 Other studies noted inadequate knowledge and lack of trust as potential contributors to vaccine hesitancy.12,13 Furthermore, vaccine hesitancy towards the influenza vaccine has been associated with vaccine hesitancy towards other critically important vaccines, such as the COVID-19 vaccine. Across various regions in China, people who have hesitated or refused to get the flu vaccine are more likely to hesitate or refuse to get the COVID-19 vaccine.14 These findings suggest that addressing vaccine hesitancy towards one disease could work synergistically towards improving the uptake of other vaccines.

Short, animated storytelling (SAS) videos are a novel and powerful approach for promoting health behaviors and communicating health messages to the general public.15,16 A specialized form of entertainment-education, SAS videos have the ability to convey evidence-based health messages by engaging audiences, simplifying complex information, eliciting emotion, and bridging cultural contexts.17,18 This wordless, culturally accessible approach was first used to scale health messages rapidly, across cultural groups, during the COVID-19 pandemic.19,20 China, with its diverse cultural and language sub-populations, poses an interesting challenge to the rapid scaling of effective health messages – a challenge that could be addressed by SAS or similar approaches. For example, a prior study, conducted in Xi'an, China, documented the significant positive effect of a 12-minute educational video aimed at promoting influenza vaccine uptake among participants over 60 years of age, living in the capital city of Shaanxi province.21 Emerging evidence from multiple studies underscores the potential of video-based interventions for increasing vaccine-related knowledge and improving vaccination outcomes.18,22,23 The feasibility of implementing this approach is also emphasized by its ease of dissemination on social media platforms. Yet, to date, no studies have explored the effect of scalable, wordless, short form (<4min) animated storytelling videos on vaccine hesitancy in China. Additionally, there is a need to explore different storytelling techniques within the SAS approach, such as humor, analogy, or emotion-driven storylines.24"

2b) In INTRODUCTION: Specific objectives or hypotheses

Does your paper address CONSORT subitem 2b? *

Copy and paste relevant sections from the manuscript (include quotes in quotation marks "like this" to indicate direct quotes from your manuscript), or elaborate on this item by providing additional information not in the ms, or briefly explain why the item is not applicable/relevant for your study

"To fill this gap, our study is the first to evaluate the effect of three SAS videos, each employing a different storytelling technique, on flu vaccine hesitancy in a diverse sample of Chinese adults. Our large-scale, nationwide, single-blind, parallel-group randomized controlled trial offers valuable insights on future design and delivery of effective SAS videos to promote health. The results of this study could also inform policy guidelines to reduce vaccine hesitancy and improve influenza outcomes across China."

METHODS

3a) Description of trial design (such as parallel, factorial) including allocation ratio

Does your paper address CONSORT subitem 3a? *

Copy and paste relevant sections from the manuscript (include quotes in quotation marks "like this" to indicate direct quotes from your manuscript), or elaborate on this item by providing additional information not in the ms, or briefly explain why the item is not applicable/relevant for your study

"We designed and implemented a nationwide single-blind, parallel-group, randomized controlled trial. "
"Participants enrolled in the study were randomly allocated to one of three video intervention groups with different storytelling techniques (humor, analogy, and emotion) or a control group in equal proportions (1:1:1:1) using a computer-generated sequence that was independent of the researchers. "

3b) Important changes to methods after trial commencement (such as eligibility criteria), with reasons

Does your paper address CONSORT subitem 3b? *

Copy and paste relevant sections from the manuscript (include quotes in quotation marks "like this" to indicate direct quotes from your manuscript), or elaborate on this item by providing additional information not in the ms, or briefly explain why the item is not applicable/relevant for your study

"We also gathered formative input from stakeholders in China, Canada, South Africa, Germany, the US, Mexico, and Australia. Our creative team, reflecting a global perspective, collaborated with international vaccine-promotion experts throughout the development process. Their feedback, shared via WhatsApp and Zoom, helped refine the video planning documents and drafts. This iterative and responsive process, recommended in human-centered design literature, ensured the interventions were continuously improved during production.34"

3b-i) Bug fixes, Downtimes, Content Changes

Bug fixes, Downtimes, Content Changes: ehealth systems are often dynamic systems. A description of changes to methods therefore also includes important changes made on the intervention or comparator during the trial (e.g., major bug fixes or changes in the functionality or content) (5-iii) and other "unexpected events" that may have influenced study design such as staff changes, system failures/downtimes, etc. [2].

	1	2	3	4	5	
subitem not at all important	○	○	○	○	○	essential

Does your paper address subitem 3b-i?

Copy and paste relevant sections from the manuscript (include quotes in quotation marks "like this" to indicate direct quotes from your manuscript), or elaborate on this item by providing additional information not in the ms, or briefly explain why the item is not applicable/relevant for your study

您的回答

4a) Eligibility criteria for participants

Does your paper address CONSORT subitem 4a? *

Copy and paste relevant sections from the manuscript (include quotes in quotation marks "like this" to indicate direct quotes from your manuscript), or elaborate on this item by providing additional information not in the ms, or briefly explain why the item is not applicable/relevant for your study

"We targeted to recruit 12,000 Chinese adults aged 18 or older, ensuring national representation through quota sampling based on age, gender, and residence (urban or rural).25-27 The quotas reflected the 2019 population estimates from the National Bureau of Statistics of China.28 Additional details is available in Text S1."

4a-i) Computer / Internet literacy

Computer / Internet literacy is often an implicit "de facto" eligibility criterion - this should be explicitly clarified.

	1	2	3	4	5	
subitem not at all important	○	○	○	○	○	essential

Does your paper address subitem 4a-i?

Copy and paste relevant sections from the manuscript (include quotes in quotation marks "like this" to indicate direct quotes from your manuscript), or elaborate on this item by providing additional information not in the ms, or briefly explain why the item is not applicable/relevant for your study

Since this video is wordless and culturally inclusive, therefore accessible to participants of all language and cultural groups.

4a-ii) Open vs. closed, web-based vs. face-to-face assessments:

Open vs. closed, web-based vs. face-to-face assessments: Mention how participants were recruited (online vs. offline), e.g., from an open access website or from a clinic, and clarify if this was a purely web-based trial, or there were face-to-face components (as part of the intervention or for assessment), i.e., to what degree got the study team to know the participant. In online-only trials, clarify if participants were quasi-anonymous and whether having multiple identities was possible or whether technical or logistical measures (e.g., cookies, email confirmation, phone calls) were used to detect/prevent these.

	1	2	3	4	5	
subitem not at all important	○	○	○	○	○	essential

Does your paper address subitem 4a-ii? *

Copy and paste relevant sections from the manuscript (include quotes in quotation marks "like this" to indicate direct quotes from your manuscript), or elaborate on this item by providing additional information not in the ms, or briefly explain why the item is not applicable/relevant for your study

"The recruitment phase of the study was conducted from April 15 to May 25, 2021. Interested participants were required to create an account on KuRunData. Upon agreeing to join our study, participants answered sociodemographic questions such as age, gender, residence type (urban or rural), education level, household annual income, and province. Intervention group participants then viewed their designated animated video intervention and completed the flu vaccine hesitancy measure (outcome measure) on either a computer or a smartphone. Control group participants filled out the questionnaire first and then were offered access to the intervention video. All procedures were completed in one session. Participants were rewarded with 5 CNY upon completing the survey."

4a-iii) Information giving during recruitment

Information given during recruitment. Specify how participants were briefed for recruitment and in the informed consent procedures (e.g., publish the informed consent documentation as appendix, see also item X26), as this information may have an effect on user self-selection, user expectation and may also bias results.

	1	2	3	4	5	
subitem not at all important	○	○	○	○	○	essential

Does your paper address subitem 4a-iii?

Copy and paste relevant sections from the manuscript (include quotes in quotation marks "like this" to indicate direct quotes from your manuscript), or elaborate on this item by providing additional information not in the ms, or briefly explain why the item is not applicable/relevant for your study

您的回答

4b) Settings and locations where the data were collected

Does your paper address CONSORT subitem 4b? *

Copy and paste relevant sections from the manuscript (include quotes in quotation marks "like this" to indicate direct quotes from your manuscript), or elaborate on this item by providing additional information not in the ms, or briefly explain why the item is not applicable/relevant for your study

"Participants were sourced through KuRunData (https://www.kurundata.com), a market research company with a membership base exceeding 17 million in China. Sampling was drawn from this large pool. Recruitment utilized various channels to achieve diverse and representative data. These channels included KuRunData's proprietary software, digital advertisements, internet searches, word of mouth, member referrals, social media platforms like TikTok, partner recommendations, and over 200 national coordinators who managed offline recruitment. This multi-source approach significantly reduced reliance on any particular demographic or group.

While they were aware this was a health-related study, potential participants were not initially informed with the specific focus of our study. After expressing their initial interest, participants received ethically approved information about the purpose and procedures of the study. Those who chose to enroll indicated their willingness by submitting an online consent form."

4b-i) Report if outcomes were (self-)assessed through online questionnaires

Clearly report if outcomes were (self-)assessed through online questionnaires (as common in web-based trials) or otherwise.

	1	2	3	4	5	
subitem not at all important	○	○	○	○	○	essential

Does your paper address subitem 4b-i? *

Copy and paste relevant sections from the manuscript (include quotes in quotation marks "like this" to indicate direct quotes from your manuscript), or elaborate on this item by providing additional information not in the ms, or briefly explain why the item is not applicable/relevant for your study

"The delivery of interventions and the collection of data were facilitated through computer webpages or smartphones."

4b-ii) Report how institutional affiliations are displayed

Report how institutional affiliations are displayed to potential participants [on ehealth media], as affiliations with prestigious hospitals or universities may affect volunteer rates, use, and reactions with regards to an intervention.(Not a required item – describe only if this may bias results)

	1	2	3	4	5	
subitem not at all important	○	○	○	○	○	essential

Does your paper address subitem 4b-ii?

Copy and paste relevant sections from the manuscript (include quotes in quotation marks "like this" to indicate direct quotes from your manuscript), or elaborate on this item by providing additional information not in the ms, or briefly explain why the item is not applicable/relevant for your study

您的回答

5) The interventions for each group with sufficient details to allow replication, including how and when they were actually administered

5-i) Mention names, credential, affiliations of the developers, sponsors, and owners

Mention names, credential, affiliations of the developers, sponsors, and owners [6] (if authors/evaluators are owners or developer of the software, this needs to be declared in a "Conflict of interest" section or mentioned elsewhere in the manuscript).

	1	2	3	4	5	
subitem not at all important	○	○	○	○	○	essential

Does your paper address subitem 5-i?

Copy and paste relevant sections from the manuscript (include quotes in quotation marks "like this" to indicate direct quotes from your manuscript), or elaborate on this item by providing additional information not in the ms, or briefly explain why the item is not applicable/relevant for your study

您的回答

5-ii) Describe the history/development process

Describe the history/development process of the application and previous formative evaluations (e.g., focus groups, usability testing), as these will have an impact on adoption/use rates and help with interpreting results.

	1	2	3	4	5	
subitem not at all important	○	○	○	○	○	essential

Does your paper address subitem 5-ii?

Copy and paste relevant sections from the manuscript (include quotes in quotation marks "like this" to indicate direct quotes from your manuscript), or elaborate on this item by providing additional information not in the ms, or briefly explain why the item is not applicable/relevant for your study

您的回答

5-iii) Revisions and updating

Revisions and updating. Clearly mention the date and/or version number of the application/intervention (and comparator, if applicable) evaluated, or describe whether the intervention underwent major changes during the evaluation process, or whether the development and/or content was “frozen” during the trial. Describe dynamic components such as news feeds or changing content which may have an impact on the replicability of the intervention (for unexpected events see item 3b).

	1	2	3	4	5	
subitem not at all important	○	○	○	○	○	essential

Does your paper address subitem 5-iii?

Copy and paste relevant sections from the manuscript (include quotes in quotation marks "like this" to indicate direct quotes from your manuscript), or elaborate on this item by providing additional information not in the ms, or briefly explain why the item is not applicable/relevant for your study

您的回答

5-iv) Quality assurance methods

Provide information on quality assurance methods to ensure accuracy and quality of information provided [1], if applicable.

	1	2	3	4	5	
subitem not at all important	○	○	○	○	○	essential

Does your paper address subitem 5-iv?

Copy and paste relevant sections from the manuscript (include quotes in quotation marks "like this" to indicate direct quotes from your manuscript), or elaborate on this item by providing additional information not in the ms, or briefly explain why the item is not applicable/relevant for your study

您的回答

5-v) Ensure replicability by publishing the source code, and/or providing screenshots/screen-capture video, and/or providing flowcharts of the algorithms used

Ensure replicability by publishing the source code, and/or providing screenshots/screen-capture video, and/or providing flowcharts of the algorithms used. Replicability (i.e., other researchers should in principle be able to replicate the study) is a hallmark of scientific reporting.

	1	2	3	4	5	
subitem not at all important	○	○	○	○	○	essential

Does your paper address subitem 5-v?

Copy and paste relevant sections from the manuscript (include quotes in quotation marks "like this" to indicate direct quotes from your manuscript), or elaborate on this item by providing additional information not in the ms, or briefly explain why the item is not applicable/relevant for your study

您的回答

5-vi) Digital preservation

Digital preservation: Provide the URL of the application, but as the intervention is likely to change or disappear over the course of the years; also make sure the intervention is archived (Internet Archive, webcitation.org, and/or publishing the source code or screenshots/videos alongside the article). As pages behind login screens cannot be archived, consider creating demo pages which are accessible without login.

	1	2	3	4	5	
subitem not at all important	○	○	○	○	○	essential

Does your paper address subitem 5-vi?

Copy and paste relevant sections from the manuscript (include quotes in quotation marks "like this" to indicate direct quotes from your manuscript), or elaborate on this item by providing additional information not in the ms, or briefly explain why the item is not applicable/relevant for your study

您的回答

5-vii) Access

Access: Describe how participants accessed the application, in what setting/context, if they had to pay (or were paid) or not, whether they had to be a member of specific group. If known, describe how participants obtained “access to the platform and Internet” [1]. To ensure access for editors/reviewers/readers, consider to provide a “backdoor” login account or demo mode for reviewers/readers to explore the application (also important for archiving purposes, see vi).

	1	2	3	4	5	
subitem not at all important	○	○	○	○	○	essential

Does your paper address subitem 5-vii? *

Copy and paste relevant sections from the manuscript (include quotes in quotation marks "like this" to indicate direct quotes from your manuscript), or elaborate on this item by providing additional information not in the ms, or briefly explain why the item is not applicable/relevant for your study

"Interested participants were required to create an account on KuRunData. Upon agreeing to join our study, participants answered sociodemographic questions such as age, gender, residence type (urban or rural), education level, household annual income, and province. Intervention group participants then viewed their designated animated video intervention and completed the flu vaccine hesitancy measure (outcome measure) on either a computer or a smartphone. Control group participants filled out the questionnaire first and then were offered access to the intervention video. All procedures were completed in one session. Participants were rewarded with 5 CNY upon completing the survey."

5-viii) Mode of delivery, features/functionalities/components of the intervention and comparator, and the theoretical framework

Describe mode of delivery, features/functionalities/components of the intervention and comparator, and the theoretical framework [6] used to design them (instructional strategy [1], behaviour change techniques, persuasive features, etc., see e.g., [7, 8] for terminology). This includes an in-depth description of the content (including where it is coming from and who developed it) [1],” whether [and how] it is tailored to individual circumstances and allows users to track their progress and receive feedback” [6]. This also includes a description of communication delivery channels and – if computer-mediated communication is a component – whether communication was synchronous or asynchronous [6]. It also includes information on presentation strategies [1], including page design principles, average amount of text on pages, presence of hyperlinks to other resources, etc. [1].

	1	2	3	4	5	
subitem not at all important	○	○	○	○	○	essential

Does your paper address subitem 5-viii? *

Copy and paste relevant sections from the manuscript (include quotes in quotation marks "like this" to indicate direct quotes from your manuscript), or elaborate on this item by providing additional information not in the ms, or briefly explain why the item is not applicable/relevant for your study

"We evaluated three short, animated entertainment-education videos, each ranging from 1 to 3 minutes in length. Video A, titled "Grandma Knows Best," employed an instructional-humor strategy29 featuring a quirky grandmother who is determined to get her family vaccinated. Video B, titled "Fly Free," utilized a storytelling-analogy approach30 in which caged birds symbolized people confined to their homes during the COVID-19 pandemic lockdowns. In this story, the birds only gained their freedom once they were vaccinated. Video C, titled "Bringing Us Together," focused on evoking emotion to connect with the audience.31 This story portrayed the widespread feelings of loneliness and isolation experienced during the pandemic lockdowns, culminating in the joy of regaining individual freedoms after vaccination. While all videos featured narratives with COVID-19 pandemic as the context, their main message was that vaccines are effective in preventing infections and promoting health. As such, they were designed to reduce vaccine hesitancy in general, including influenza vaccine hesitancy. Figure 1 displays selected screenshots from these interventions with each video employing a unique instructional storytelling technique (i.e., humor, analogy and emotion).

The production of the intervention videos took place between November 2020 and March 2021 during the initial distribution of the first COVID-19 vaccines. We collaborated with an interdisciplinary team of experts, including those specializing in behavioral sciences, entertainment, and marketing. This approach allowed us to incorporate evidence-based vaccine-promotion messages into engaging and scalable short, animated entertainment-education videos. The character designs were culturally inclusive and based on insights from our previous study, which involved participants from 73 countries.32

We utilized the principles of Universal Design for Learning (UDL) to ensure the inclusiveness of the videos, with each of the three videos employing slightly different methods.33 Video A used iconic character representations without cultural identifiers and different shades of grey to indicate racial diversity. Video B illustrated diversity through various bird species. Video C adopted a hybrid approach, featuring an icon-style main character without cultural markers, with supporting characters representing diverse races and ethnicities. We also gathered formative input from stakeholders in China, Canada, South Africa, Germany, the US, Mexico, and Australia. Our creative team, reflecting a global perspective, collaborated with international vaccine-promotion experts throughout the development process. Their feedback, shared via WhatsApp and Zoom, helped refine the video planning documents and drafts. This iterative and responsive process, recommended in human-centered design literature, ensured the interventions were continuously improved during production.34

All three intervention videos were created in the same 2D animation style by the same animator. To maximize scalability across different language speakers, none of the videos included spoken language. This decision was influenced by the observed spontaneous distribution of our earlier wordless, animated entertainment-education content.20 Instead of dialogue, each video relied on visual storytelling, complemented by an engaging soundtrack. The intervention videos can be accessed on YouTube via the following links:
- Video A (humor): https://youtu.be/ap8xpyREaTc
- Video B (analogy): https://youtu.be/fYYBJ0d6gl0
- Video C (emotion): https://youtu.be/WH5KUhGtfa8"

5-ix) Describe use parameters

Describe use parameters (e.g., intended “doses” and optimal timing for use). Clarify what instructions or recommendations were given to the user, e.g., regarding timing, frequency, heaviness of use, if any, or was the intervention used ad libitum.

	1	2	3	4	5	
subitem not at all important	○	○	○	○	○	essential

Does your paper address subitem 5-ix?

Copy and paste relevant sections from the manuscript (include quotes in quotation marks "like this" to indicate direct quotes from your manuscript), or elaborate on this item by providing additional information not in the ms, or briefly explain why the item is not applicable/relevant for your study

您的回答

5-x) Clarify the level of human involvement

Clarify the level of human involvement (care providers or health professionals, also technical assistance) in the e-intervention or as co-intervention (detail number and expertise of professionals involved, if any, as well as “type of assistance offered, the timing and frequency of the support, how it is initiated, and the medium by which the assistance is delivered”. It may be necessary to distinguish between the level of human involvement required for the trial, and the level of human involvement required for a routine application outside of a RCT setting (discuss under item 21 – generalizability).

	1	2	3	4	5	
subitem not at all important	○	○	○	○	○	essential

Does your paper address subitem 5-x?

Copy and paste relevant sections from the manuscript (include quotes in quotation marks "like this" to indicate direct quotes from your manuscript), or elaborate on this item by providing additional information not in the ms, or briefly explain why the item is not applicable/relevant for your study

您的回答

5-xi) Report any prompts/reminders used

Report any prompts/reminders used: Clarify if there were prompts (letters, emails, phone calls, SMS) to use the application, what triggered them, frequency etc. It may be necessary to distinguish between the level of prompts/reminders required for the trial, and the level of prompts/reminders for a routine application outside of a RCT setting (discuss under item 21 – generalizability).

	1	2	3	4	5	
subitem not at all important	○	○	○	○	○	essential

Does your paper address subitem 5-xi? *

Copy and paste relevant sections from the manuscript (include quotes in quotation marks "like this" to indicate direct quotes from your manuscript), or elaborate on this item by providing additional information not in the ms, or briefly explain why the item is not applicable/relevant for your study

In the context of our study, prompts and reminders were not utilized. The intervention involved participants watching videos online, after which the assessment of the intervention's impact was conducted immediately. Therefore, there was no need for any prompts or reminders to encourage ongoing or repeated engagement with the intervention.

5-xii) Describe any co-interventions (incl. training/support)

Describe any co-interventions (incl. training/support): Clearly state any interventions that are provided in addition to the targeted eHealth intervention, as ehealth intervention may not be designed as stand-alone intervention. This includes training sessions and support [1]. It may be necessary to distinguish between the level of training required for the trial, and the level of training for a routine application outside of a RCT setting (discuss under item 21 – generalizability.

	1	2	3	4	5	
subitem not at all important	○	○	○	○	○	essential

Does your paper address subitem 5-xii? *

Copy and paste relevant sections from the manuscript (include quotes in quotation marks "like this" to indicate direct quotes from your manuscript), or elaborate on this item by providing additional information not in the ms, or briefly explain why the item is not applicable/relevant for your study

In our study, no co-interventions, including training sessions or additional support, were provided alongside the targeted eHealth intervention. The intervention was designed to be as scalable and straightforward as possible, aiming to maximize its effectiveness through simplicity. We intended for the intervention to be immediately applicable and self-explanatory, requiring no additional training or support to understand or engage with the content. This approach enhances the potential scalability of the intervention, as it can be deployed broadly without the need for extensive resources or preparations typically associated with training and support mechanisms. Thus, discussing the levels of training for routine applications outside of an RCT setting is not relevant for this specific study.

6a) Completely defined pre-specified primary and secondary outcome measures, including how and when they were assessed

Does your paper address CONSORT subitem 6a? *

Copy and paste relevant sections from the manuscript (include quotes in quotation marks "like this" to indicate direct quotes from your manuscript), or elaborate on this item by providing additional information not in the ms, or briefly explain why the item is not applicable/relevant for your study

"To assess participants' hesitancy towards the influenza vaccine, we utilized the validated Adult Vaccine Hesitancy Scale (aVHS), which has been verified for the Chinese adult population.35,36 Derived from the 14-item WHO SAGE Vaccine Hesitancy Scale, the aVHS consists of 10 items, including 3 negative and 7 positive (reverse-coded) statements.37 Participants rated their responses for each item on a five-point scale, producing total scores ranging from 10 to 50, with higher scores indicating greater vaccine hesitancy."

6a-i) Online questionnaires: describe if they were validated for online use and apply CHERRIES items to describe how the questionnaires were designed/deployed

If outcomes were obtained through online questionnaires, describe if they were validated for online use and apply CHERRIES items to describe how the questionnaires were designed/deployed [9].

	1	2	3	4	5	
subitem not at all important	○	○	○	○	○	essential

Does your paper address subitem 6a-i?

Copy and paste relevant sections from manuscript text

您的回答

6a-ii) Describe whether and how “use” (including intensity of use/dosage) was defined/measured/monitored

Describe whether and how “use” (including intensity of use/dosage) was defined/measured/monitored (logins, logfile analysis, etc.). Use/adoption metrics are important process outcomes that should be reported in any ehealth trial.

	1	2	3	4	5	
subitem not at all important	○	○	○	○	○	essential

Does your paper address subitem 6a-ii?

Copy and paste relevant sections from manuscript text

您的回答

6a-iii) Describe whether, how, and when qualitative feedback from participants was obtained

Describe whether, how, and when qualitative feedback from participants was obtained (e.g., through emails, feedback forms, interviews, focus groups).

	1	2	3	4	5	
subitem not at all important	○	○	○	○	○	essential

Does your paper address subitem 6a-iii?

Copy and paste relevant sections from manuscript text

您的回答

6b) Any changes to trial outcomes after the trial commenced, with reasons

Does your paper address CONSORT subitem 6b? *

Copy and paste relevant sections from the manuscript (include quotes in quotation marks "like this" to indicate direct quotes from your manuscript), or elaborate on this item by providing additional information not in the ms, or briefly explain why the item is not applicable/relevant for your study

There were no changes to the trial outcomes after the trial commenced. The outcomes defined at the outset of the study were maintained throughout the duration of the trial without any modifications. This consistency was maintained to ensure the integrity of the study and to accurately measure the effectiveness of the intervention as originally planned.

7a) How sample size was determined

NPT: When applicable, details of whether and how the clustering by care provides or centers was addressed

7a-i) Describe whether and how expected attrition was taken into account when calculating the sample size

Describe whether and how expected attrition was taken into account when calculating the sample size.

	1	2	3	4	5	
subitem not at all important	○	○	○	○	○	essential

Does your paper address subitem 7a-i?

Copy and paste relevant sections from manuscript title (include quotes in quotation marks "like this" to indicate direct quotes from your manuscript), or elaborate on this item by providing additional information not in the ms, or briefly explain why the item is not applicable/relevant for your study

您的回答

7b) When applicable, explanation of any interim analyses and stopping guidelines

Does your paper address CONSORT subitem 7b? *

Copy and paste relevant sections from the manuscript (include quotes in quotation marks "like this" to indicate direct quotes from your manuscript), or elaborate on this item by providing additional information not in the ms, or briefly explain why the item is not applicable/relevant for your study

In our study, there were no interim analyses or specific stopping guidelines implemented. The design of our trial did not require these elements as it was structured to complete as planned without the need for interim evaluation points. The duration and scope of the trial were set to allow a straightforward assessment of outcomes upon its conclusion, ensuring that comprehensive data was collected without the necessity for early analysis or premature termination guidelines. This approach was aligned with the study's objectives to measure the effects of the intervention in a direct and uninterrupted manner.

8a) Method used to generate the random allocation sequence

NPT: When applicable, how care providers were allocated to each trial group

Does your paper address CONSORT subitem 8a? *

Copy and paste relevant sections from the manuscript (include quotes in quotation marks "like this" to indicate direct quotes from your manuscript), or elaborate on this item by providing additional information not in the ms, or briefly explain why the item is not applicable/relevant for your study

"Participants enrolled in the study were randomly allocated to one of three video intervention groups with different storytelling techniques (humor, analogy, and emotion) or a control group in equal proportions (1:1:1:1) using a computer-generated sequence that was independent of the researchers. Those in the intervention groups viewed one of the three intervention videos, without being informed of the other conditions. Each participant was assigned a unique, anonymous ID and required to complete a series of validated surveys to collect data. Data was gathered by KuRunData, ensuring participant anonymity from the research team, which only accessed deidentified data during the analysis phase. Throughout the trial and data analysis, the research team remained blinded to the specific assignments of individual participants."

8b) Type of randomisation; details of any restriction (such as blocking and block size)

Does your paper address CONSORT subitem 8b? *

Copy and paste relevant sections from the manuscript (include quotes in quotation marks "like this" to indicate direct quotes from your manuscript), or elaborate on this item by providing additional information not in the ms, or briefly explain why the item is not applicable/relevant for your study

"Participants enrolled in the study were randomly allocated to one of three video intervention groups with different storytelling techniques (humor, analogy, and emotion) or a control group in equal proportions (1:1:1:1) using a computer-generated sequence that was independent of the researchers. Those in the intervention groups viewed one of the three intervention videos, without being informed of the other conditions. Each participant was assigned a unique, anonymous ID and required to complete a series of validated surveys to collect data. Data was gathered by KuRunData, ensuring participant anonymity from the research team, which only accessed deidentified data during the analysis phase. Throughout the trial and data analysis, the research team remained blinded to the specific assignments of individual participants."

9) Mechanism used to implement the random allocation sequence (such as sequentially numbered containers), describing any steps taken to conceal the sequence until interventions were assigned

Does your paper address CONSORT subitem 9? *

Copy and paste relevant sections from the manuscript (include quotes in quotation marks "like this" to indicate direct quotes from your manuscript), or elaborate on this item by providing additional information not in the ms, or briefly explain why the item is not applicable/relevant for your study

"Participants enrolled in the study were randomly allocated to one of three video intervention groups with different storytelling techniques (humor, analogy, and emotion) or a control group in equal proportions (1:1:1:1) using a computer-generated sequence that was independent of the researchers. Those in the intervention groups viewed one of the three intervention videos, without being informed of the other conditions. Each participant was assigned a unique, anonymous ID and required to complete a series of validated surveys to collect data. Data was gathered by KuRunData, ensuring participant anonymity from the research team, which only accessed deidentified data during the analysis phase. Throughout the trial and data analysis, the research team remained blinded to the specific assignments of individual participants."

10) Who generated the random allocation sequence, who enrolled participants, and who assigned participants to interventions

Does your paper address CONSORT subitem 10? *

Copy and paste relevant sections from the manuscript (include quotes in quotation marks "like this" to indicate direct quotes from your manuscript), or elaborate on this item by providing additional information not in the ms, or briefly explain why the item is not applicable/relevant for your study

"Participants enrolled in the study were randomly allocated to one of three video intervention groups with different storytelling techniques (humor, analogy, and emotion) or a control group in equal proportions (1:1:1:1) using a computer-generated sequence that was independent of the researchers. Those in the intervention groups viewed one of the three intervention videos, without being informed of the other conditions. Each participant was assigned a unique, anonymous ID and required to complete a series of validated surveys to collect data. Data was gathered by KuRunData, ensuring participant anonymity from the research team, which only accessed deidentified data during the analysis phase. Throughout the trial and data analysis, the research team remained blinded to the specific assignments of individual participants."

11a) If done, who was blinded after assignment to interventions (for example, participants, care providers, those assessing outcomes) and how

NPT: Whether or not administering co-interventions were blinded to group assignment

11a-i) Specify who was blinded, and who wasn’t

Specify who was blinded, and who wasn’t. Usually, in web-based trials it is not possible to blind the participants [1, 3] (this should be clearly acknowledged), but it may be possible to blind outcome assessors, those doing data analysis or those administering co-interventions (if any).

	1	2	3	4	5	
subitem not at all important	○	○	○	○	○	essential

Does your paper address subitem 11a-i? *

Copy and paste relevant sections from the manuscript (include quotes in quotation marks "like this" to indicate direct quotes from your manuscript), or elaborate on this item by providing additional information not in the ms, or briefly explain why the item is not applicable/relevant for your study

"Those in the intervention groups viewed one of the three intervention videos, without being informed of the other conditions. Each participant was assigned a unique, anonymous ID and required to complete a series of validated surveys to collect data. Data was gathered by KuRunData, ensuring participant anonymity from the research team, which only accessed deidentified data during the analysis phase. Throughout the trial and data analysis, the research team remained blinded to the specific assignments of individual participants."

11a-ii) Discuss e.g., whether participants knew which intervention was the "intervention of interest" and which one was the "comparator"

Informed consent procedures (4a-ii) can create biases and certain expectations - discuss e.g., whether participants knew which intervention was the "intervention of interest" and which one was the "comparator".

	1	2	3	4	5	
subitem not at all important	○	○	○	○	○	essential

Does your paper address subitem 11a-ii?

Copy and paste relevant sections from the manuscript (include quotes in quotation marks "like this" to indicate direct quotes from your manuscript), or elaborate on this item by providing additional information not in the ms, or briefly explain why the item is not applicable/relevant for your study

您的回答

11b) If relevant, description of the similarity of interventions

(this item is usually not relevant for ehealth trials as it refers to similarity of a placebo or sham intervention to a active medication/intervention)

Does your paper address CONSORT subitem 11b? *

Copy and paste relevant sections from the manuscript (include quotes in quotation marks "like this" to indicate direct quotes from your manuscript), or elaborate on this item by providing additional information not in the ms, or briefly explain why the item is not applicable/relevant for your study

This item is not applicable to our study as our trial did not involve placebo or sham interventions typically used in clinical trials involving active medications. Our study focused on digital health interventions where the primary comparison was between different types of content delivery or no intervention at all. Therefore, the concept of similarity, as it pertains to placebo or sham equivalents, does not apply.

12a) Statistical methods used to compare groups for primary and secondary outcomes

NPT: When applicable, details of whether and how the clustering by care providers or centers was addressed

Does your paper address CONSORT subitem 12a? *

Copy and paste relevant sections from the manuscript (include quotes in quotation marks "like this" to indicate direct quotes from your manuscript), or elaborate on this item by providing additional information not in the ms, or briefly explain why the item is not applicable/relevant for your study

"Baseline characteristics included age, gender, residence, education level, household annual income, region, and economic belt, which were summarized by means (SDs) or n/N (%) as appropriate. To further differentiate the regions of China by their socioeconomic development status, we divided all regions into four economic belts, namely, East, Central, West, and Northeast.38 We applied Pearson's Chi-squared test (with categorical variables) and Kruskal-Wallis rank sum test (with continuous variables) to compare baseline characteristics across groups. The primary analysis adhered to the intention-to-treat (ITT) principle. First, a pooled analysis combined participants from the three intervention groups and compared them with the control group using independent t-tests for two-tailed p-values. Secondly, we evaluated flu vaccine hesitancy through six predefined co-primary comparisons (each intervention group versus the control group, and comparisons between intervention groups). Bonferroni correction was applied to adjust for multiple pairwise comparisons.39 The overall alpha level of 0.05 was evenly distributed across all co-primary comparisons; therefore, only p-values (pheterogeneity) <0.0083 were considered statistically significant.40 For each co-primary comparison, we calculated the absolute difference in means and Cohen's effect size d (Cohen's d) along with nominal 99.17% confidence intervals (CIs) to maintain an overall alpha level of 0.05.

With a target of 5% type I error rate (two-sided) and a 20% type II error rate, a minimum sample size of 6,280 was required to detect a 0.01 difference in mean vaccine hesitancy scores between groups (1 = total acceptance, 5 = complete refusal), assuming a standard deviation of 0.10. We recruited 12,000 participants to account for multiple comparison adjustments. Given the extremely short duration of the interventions tested in this trial (1to 3 minutes single exposures), enhanced statistical power was deemed necessary to detect any potential effects of these “micro-interventions.” 41-43 Detailed calculations are provided in the study protocol.24

Subgroup analyses were conducted for each predefined subgroup by baseline characteristics, with interaction p-values calculated using two-tailed large sample Z tests.44 Interaction p-values (pinteraction) below 0.05 were considered statistically significant. We differentiated between interaction effects, which examine significant differences in intervention effectiveness across subgroups, and heterogeneity, which indicates variability in effectiveness within subgroups.45 All data and statistical analyses were performed in R (version 4.1.2)."

12a-i) Imputation techniques to deal with attrition / missing values

Imputation techniques to deal with attrition / missing values: Not all participants will use the intervention/comparator as intended and attrition is typically high in ehealth trials. Specify how participants who did not use the application or dropped out from the trial were treated in the statistical analysis (a complete case analysis is strongly discouraged, and simple imputation techniques such as LOCF may also be problematic [4]).

	1	2	3	4	5	
subitem not at all important	○	○	○	○	○	essential

Does your paper address subitem 12a-i? *

Copy and paste relevant sections from the manuscript (include quotes in quotation marks "like this" to indicate direct quotes from your manuscript), or elaborate on this item by providing additional information not in the ms, or briefly explain why the item is not applicable/relevant for your study

In our study, we used reaction times to identify participants who were not sufficiently engaged, i.e., those who did not watch the intervention videos or respond to the questions seriously. Participants with unusually quick or long reaction times, suggesting a lack of proper engagement, were directly excluded from the dataset. This approach was chosen because our intervention videos were very short (1-3 minutes), and thus, the overall compliance among those who remained in the study was high.

12b) Methods for additional analyses, such as subgroup analyses and adjusted analyses

Does your paper address CONSORT subitem 12b? *

Copy and paste relevant sections from the manuscript (include quotes in quotation marks "like this" to indicate direct quotes from your manuscript), or elaborate on this item by providing additional information not in the ms, or briefly explain why the item is not applicable/relevant for your study

"Subgroup analyses were conducted for each predefined subgroup by baseline characteristics, with interaction p-values calculated using two-tailed large sample Z tests.44 Interaction p-values (pinteraction) below 0.05 were considered statistically significant. We differentiated between interaction effects, which examine significant differences in intervention effectiveness across subgroups, and heterogeneity, which indicates variability in effectiveness within subgroups.45 All data and statistical analyses were performed in R (version 4.1.2)."

X26) REB/IRB Approval and Ethical Considerations [recommended as subheading under "Methods"] (not a CONSORT item)

X26-i) Comment on ethics committee approval

	1	2	3	4	5	
subitem not at all important	○	○	○	○	○	essential

Does your paper address subitem X26-i?

Copy and paste relevant sections from the manuscript (include quotes in quotation marks "like this" to indicate direct quotes from your manuscript), or elaborate on this item by providing additional information not in the ms, or briefly explain why the item is not applicable/relevant for your study

您的回答

x26-ii) Outline informed consent procedures

Outline informed consent procedures e.g., if consent was obtained offline or online (how? Checkbox, etc.?), and what information was provided (see 4a-ii). See [6] for some items to be included in informed consent documents.

	1	2	3	4	5	
subitem not at all important	○	○	○	○	○	essential

Does your paper address subitem X26-ii?

Copy and paste relevant sections from the manuscript (include quotes in quotation marks "like this" to indicate direct quotes from your manuscript), or elaborate on this item by providing additional information not in the ms, or briefly explain why the item is not applicable/relevant for your study

您的回答

X26-iii) Safety and security procedures

Safety and security procedures, incl. privacy considerations, and any steps taken to reduce the likelihood or detection of harm (e.g., education and training, availability of a hotline)

	1	2	3	4	5	
subitem not at all important	○	○	○	○	○	essential

Does your paper address subitem X26-iii?

Copy and paste relevant sections from the manuscript (include quotes in quotation marks "like this" to indicate direct quotes from your manuscript), or elaborate on this item by providing additional information not in the ms, or briefly explain why the item is not applicable/relevant for your study

您的回答

RESULTS

13a) For each group, the numbers of participants who were randomly assigned, received intended treatment, and were analysed for the primary outcome
NPT: The number of care providers or centers performing the intervention in each group and the number of patients treated by each care provider in each center

Does your paper address CONSORT subitem 13a? *

Copy and paste relevant sections from the manuscript (include quotes in quotation marks "like this" to indicate direct quotes from your manuscript), or elaborate on this item by providing additional information not in the ms, or briefly explain why the item is not applicable/relevant for your study

"Figure S1: Study flowchart"
"A total of 18,422 individuals indicated their interest in the study. Among them, 2,279 (12.37%) were excluded for failing to submit the questionnaires, 939 (5.10%) were excluded for being under 18 years of age or not living in China, and 2,555 (13.87%) were excluded due to reaching full participant quotas. As a result, 12,649 (68.66%) individuals met the initial eligibility criteria and were randomly assigned to intervention and control groups. Of these, 649 individuals were excluded because of insufficient response times or issues with backup data (randomly selected from participants beyond the required 12,000 to replace any low-quality data), leaving 12,000 participants (65.14%) included in the final analysis (Figure S1).
"

13b) For each group, losses and exclusions after randomisation, together with reasons

Does your paper address CONSORT subitem 13b? (NOTE: Preferably, this is shown in a CONSORT flow diagram) *

Copy and paste relevant sections from the manuscript (include quotes in quotation marks "like this" to indicate direct quotes from your manuscript), or elaborate on this item by providing additional information not in the ms, or briefly explain why the item is not applicable/relevant for your study

"Figure S1: Study flowchart"
"A total of 18,422 individuals indicated their interest in the study. Among them, 2,279 (12.37%) were excluded for failing to submit the questionnaires, 939 (5.10%) were excluded for being under 18 years of age or not living in China, and 2,555 (13.87%) were excluded due to reaching full participant quotas. As a result, 12,649 (68.66%) individuals met the initial eligibility criteria and were randomly assigned to intervention and control groups. Of these, 649 individuals were excluded because of insufficient response times or issues with backup data (randomly selected from participants beyond the required 12,000 to replace any low-quality data), leaving 12,000 participants (65.14%) included in the final analysis (Figure S1).
"

13b-i) Attrition diagram

Strongly recommended: An attrition diagram (e.g., proportion of participants still logging in or using the intervention/comparator in each group plotted over time, similar to a survival curve) or other figures or tables demonstrating usage/dose/engagement.

	1	2	3	4	5	
subitem not at all important	○	○	○	○	○	essential

Does your paper address subitem 13b-i?

Copy and paste relevant sections from the manuscript or cite the figure number if applicable (include quotes in quotation marks "like this" to indicate direct quotes from your manuscript), or elaborate on this item by providing additional information not in the ms, or briefly explain why the item is not applicable/relevant for your study

您的回答

14a) Dates defining the periods of recruitment and follow-up

Does your paper address CONSORT subitem 14a? *

Copy and paste relevant sections from the manuscript (include quotes in quotation marks "like this" to indicate direct quotes from your manuscript), or elaborate on this item by providing additional information not in the ms, or briefly explain why the item is not applicable/relevant for your study

"The recruitment phase of the study was conducted from April 15 to May 25, 2021."

14a-i) Indicate if critical “secular events” fell into the study period

Indicate if critical “secular events” fell into the study period, e.g., significant changes in Internet resources available or “changes in computer hardware or Internet delivery resources”

	1	2	3	4	5	
subitem not at all important	○	○	○	○	○	essential

Does your paper address subitem 14a-i?

Copy and paste relevant sections from the manuscript (include quotes in quotation marks "like this" to indicate direct quotes from your manuscript), or elaborate on this item by providing additional information not in the ms, or briefly explain why the item is not applicable/relevant for your study

您的回答

14b) Why the trial ended or was stopped (early)

Does your paper address CONSORT subitem 14b? *

Copy and paste relevant sections from the manuscript (include quotes in quotation marks "like this" to indicate direct quotes from your manuscript), or elaborate on this item by providing additional information not in the ms, or briefly explain why the item is not applicable/relevant for your study

This item is not applicable to our study. The trial was completed as originally planned without any early termination. All participants completed the study within the designated timeframe, and no issues arose that required the trial to be stopped early.

15) A table showing baseline demographic and clinical characteristics for each group

NPT: When applicable, a description of care providers (case volume, qualification, expertise, etc.) and centers (volume) in each group

Does your paper address CONSORT subitem 15? *

Copy and paste relevant sections from the manuscript (include quotes in quotation marks "like this" to indicate direct quotes from your manuscript), or elaborate on this item by providing additional information not in the ms, or briefly explain why the item is not applicable/relevant for your study

"A total of 18,422 individuals indicated their interest in the study. Among them, 2,279 (12.37%) were excluded for failing to submit the questionnaires, 939 (5.10%) were excluded for being under 18 years of age or not living in China, and 2,555 (13.87%) were excluded due to reaching full participant quotas. As a result, 12,649 (68.66%) individuals met the initial eligibility criteria and were randomly assigned to intervention and control groups. Of these, 649 individuals were excluded because of insufficient response times or issues with backup data (randomly selected from participants beyond the required 12,000 to replace any low-quality data), leaving 12,000 participants (65.14%) included in the final analysis (Figure S1). In the final study cohort, the participants resided in 31 of the 32 provinces, autonomous regions, and municipalities of mainland China. (Figure S2). The average age of the participants was 44.3 years (SD 14.3), with 5,873 (48.9%) women and 6,127 (51.1%) men. Among them, 4,744 (39.5%) lived in rural areas, 3,600 (30.0%) had junior high or lower educational levels, 4,411 (36.8%) had annual family incomes under 90,000 CNY, 3,680 (30.67%) were from the western region, and 4,440 (37.0%) were from the western economic belt. The baseline characteristics were balanced across the intervention and control groups (Table 1). Additionally, data from the study period revealed that only 5 participants (0.04%) had a history of COVID-19."

15-i) Report demographics associated with digital divide issues

In ehealth trials it is particularly important to report demographics associated with digital divide issues, such as age, education, gender, social-economic status, computer/Internet/ehealth literacy of the participants, if known.

	1	2	3	4	5	
subitem not at all important	○	○	○	○	○	essential

Does your paper address subitem 15-i? *

Copy and paste relevant sections from the manuscript (include quotes in quotation marks "like this" to indicate direct quotes from your manuscript), or elaborate on this item by providing additional information not in the ms, or briefly explain why the item is not applicable/relevant for your study

"The average age of the participants was 44.3 years (SD 14.3), with 5,873 (48.9%) women and 6,127 (51.1%) men. Among them, 4,744 (39.5%) lived in rural areas, 3,600 (30.0%) had junior high or lower educational levels, 4,411 (36.8%) had annual family incomes under 90,000 CNY, 3,680 (30.67%) were from the western region, and 4,440 (37.0%) were from the western economic belt. The baseline characteristics were balanced across the intervention and control groups (Table 1). Additionally, data from the study period revealed that only 5 participants (0.04%) had a history of COVID-19."

16) For each group, number of participants (denominator) included in each analysis and whether the analysis was by original assigned groups

16-i) Report multiple "denominators" and provide definitions

Report multiple "denominators" and provide definitions: Report N's (and effect sizes) "across a range of study participation [and use] thresholds" [1], e.g., N exposed, N consented, N used more than x times, N used more than y weeks, N participants "used" the intervention/comparator at specific pre-defined time points of interest (in absolute and relative numbers per group). Always clearly define "use" of the intervention.

	1	2	3	4	5	
subitem not at all important	○	○	○	○	○	essential

Does your paper address subitem 16-i? *

Copy and paste relevant sections from the manuscript (include quotes in quotation marks "like this" to indicate direct quotes from your manuscript), or elaborate on this item by providing additional information not in the ms, or briefly explain why the item is not applicable/relevant for your study

"Table 1: Baseline characteristics across randomization groups in total study population"; "Figure 2: Effects of animated videos on flu vaccine hesitancy"; "Figure 3: Effects of animated video A on flu vaccine hesitancy across prespecified subgroups"; "Figure 4: Effects of animated video B on flu vaccine hesitancy across prespecified subgroups"; "Figure 5: Effects of animated video C on flu vaccine hesitancy across prespecified subgroups"

16-ii) Primary analysis should be intent-to-treat

Primary analysis should be intent-to-treat, secondary analyses could include comparing only "users", with the appropriate caveats that this is no longer a randomized sample (see 18-i).

	1	2	3	4	5	
subitem not at all important	○	○	○	○	○	essential

Does your paper address subitem 16-ii?

Copy and paste relevant sections from the manuscript (include quotes in quotation marks "like this" to indicate direct quotes from your manuscript), or elaborate on this item by providing additional information not in the ms, or briefly explain why the item is not applicable/relevant for your study

您的回答

17a) For each primary and secondary outcome, results for each group, and the estimated effect size and its precision (such as 95% confidence interval)

Does your paper address CONSORT subitem 17a? *

Copy and paste relevant sections from the manuscript (include quotes in quotation marks "like this" to indicate direct quotes from your manuscript), or elaborate on this item by providing additional information not in the ms, or briefly explain why the item is not applicable/relevant for your study

"Overall effectiveness of SAS intervention
In the main analysis, participants in any of the three intervention groups showed lower flu vaccine hesitancy compared with the control group, with a mean difference of -0.41 (95% CI: -0.60 to -0.23; p-value < 0.0001) measured in aVHS scores. Specifically, participants who received Video A intervention showed significantly lower flu vaccine hesitancy than the control group (-0.44, 99.17% CI -0.75 to -0.13; p-value = 0.0002); those who received intervention with Video B also showed lower hesitancy compared to the control group (-0.55, 99.17% CI: -0.86 to -0.24; p-value < 0.0001; Figure 2). However, no significant difference in the flu vaccination hesitancy was observed between the interventions groups with Video A (humor) vs. Video B (analogy). In addition, no significant effects were observed in the Video C (emotion) group compared to the control group. Similarly, no significant differences were found when comparing Video C (emotion) with other intervention groups with either Video A (humor) or Video B (analogy) (Figure 2)."

17a-i) Presentation of process outcomes such as metrics of use and intensity of use

In addition to primary/secondary (clinical) outcomes, the presentation of process outcomes such as metrics of use and intensity of use (dose, exposure) and their operational definitions is critical. This does not only refer to metrics of attrition (13-b) (often a binary variable), but also to more continuous exposure metrics such as “average session length”. These must be accompanied by a technical description how a metric like a “session” is defined (e.g., timeout after idle time) [1] (report under item 6a).

	1	2	3	4	5	
subitem not at all important	○	○	○	○	○	essential

Does your paper address subitem 17a-i?

Copy and paste relevant sections from the manuscript (include quotes in quotation marks "like this" to indicate direct quotes from your manuscript), or elaborate on this item by providing additional information not in the ms, or briefly explain why the item is not applicable/relevant for your study

您的回答

17b) For binary outcomes, presentation of both absolute and relative effect sizes is recommended

Does your paper address CONSORT subitem 17b? *

Copy and paste relevant sections from the manuscript (include quotes in quotation marks "like this" to indicate direct quotes from your manuscript), or elaborate on this item by providing additional information not in the ms, or briefly explain why the item is not applicable/relevant for your study

This item is not applicable to our study as it does not involve binary outcomes.

18) Results of any other analyses performed, including subgroup analyses and adjusted analyses, distinguishing pre-specified from exploratory

Does your paper address CONSORT subitem 18? *

Copy and paste relevant sections from the manuscript (include quotes in quotation marks "like this" to indicate direct quotes from your manuscript), or elaborate on this item by providing additional information not in the ms, or briefly explain why the item is not applicable/relevant for your study

"Subgroup analysis
Subgroup analyses were performed for the comparisons between intervention group Video A (humor) vs. control group, Video B (analogy) vs. control group, and Video C (emotion) vs. control group, respectively. The forest plots illustrating the effectiveness of each animated video compared to the control group, moderated by factors such as age, gender, residence, education level, household annual income, region, and economic belt, are presented in Figures 3-5. In these subgroup analyses, a negative mean difference implies that the intervention group had lower hesitancy scores than the control group, meaning a positive effect of the intervention on reducing the flu vaccination hesitancy.

In particular, the intervention group with Video A (humor) showed significantly lower hesitancy compared to the control group when stratified by residence and region. Participants in urban areas (-0.66, 99.17% CI: -1.06 to -0.26; pinteraction = 0.0252), the South (-1.40, 99.17% CI: -2.35 to -0.44), and the Southwest (-1.17, 99.17% CI: -1.97 to -0.37; pinteraction = 0.0072) exhibited greater reductions in flu vaccine hesitancy than the control group. Additionally, significant heterogeneity was observed within subgroups of males, urban residents, households with an income of 90,000-180,000 CNY, the south and southwest region, and the western economic belt (pheterogeneity < 0.0083), indicating that Video A reduced vaccine hesitancy within these subgroups. Although Video B significantly reduced flu vaccine hesitancy overall compared with control group, the interaction effects between subgroups were not significant (pinteraction > 0.05), indicating no significant differences in intervention effects among different subgroups. However, significant heterogeneity was observed within certain subgroups, including those aged 40-49 years, males, females, rural residents, urban residents, participants with a college education or higher, households with an income of 90,000-180,000 CNY, the southwest region, and the western economic belt (pheterogeneity < 0.0083), indicating that Video B reduced vaccine hesitancy within these subgroups. Video C did not show a significant overall effect or differences by subgroup "

18-i) Subgroup analysis of comparing only users

A subgroup analysis of comparing only users is not uncommon in ehealth trials, but if done, it must be stressed that this is a self-selected sample and no longer an unbiased sample from a randomized trial (see 16-iii).

	1	2	3	4	5	
subitem not at all important	○	○	○	○	○	essential

Does your paper address subitem 18-i?

Copy and paste relevant sections from the manuscript (include quotes in quotation marks "like this" to indicate direct quotes from your manuscript), or elaborate on this item by providing additional information not in the ms, or briefly explain why the item is not applicable/relevant for your study

您的回答

19) All important harms or unintended effects in each group
(for specific guidance see CONSORT for harms)

Does your paper address CONSORT subitem 19? *

Copy and paste relevant sections from the manuscript (include quotes in quotation marks "like this" to indicate direct quotes from your manuscript), or elaborate on this item by providing additional information not in the ms, or briefly explain why the item is not applicable/relevant for your study

"As this is an online survey, there is very minimal risk for study-related injury or harm. We will present nonthreatening video interventions and questions about vaccine attitudes. There are no foreseeable risks to participating in the online study."

19-i) Include privacy breaches, technical problems

Include privacy breaches, technical problems. This does not only include physical “harm” to participants, but also incidents such as perceived or real privacy breaches [1], technical problems, and other unexpected/unintended incidents. “Unintended effects” also includes unintended positive effects [2].

	1	2	3	4	5	
subitem not at all important	○	○	○	○	○	essential

Does your paper address subitem 19-i?

Copy and paste relevant sections from the manuscript (include quotes in quotation marks "like this" to indicate direct quotes from your manuscript), or elaborate on this item by providing additional information not in the ms, or briefly explain why the item is not applicable/relevant for your study

您的回答

19-ii) Include qualitative feedback from participants or observations from staff/researchers

Include qualitative feedback from participants or observations from staff/researchers, if available, on strengths and shortcomings of the application, especially if they point to unintended/unexpected effects or uses. This includes (if available) reasons for why people did or did not use the application as intended by the developers.

	1	2	3	4	5	
subitem not at all important	○	○	○	○	○	essential

Does your paper address subitem 19-ii?

Copy and paste relevant sections from the manuscript (include quotes in quotation marks "like this" to indicate direct quotes from your manuscript), or elaborate on this item by providing additional information not in the ms, or briefly explain why the item is not applicable/relevant for your study

您的回答

DISCUSSION

22) Interpretation consistent with results, balancing benefits and harms, and considering other relevant evidence

NPT: In addition, take into account the choice of the comparator, lack of or partial blinding, and unequal expertise of care providers or centers in each group

22-i) Restate study questions and summarize the answers suggested by the data, starting with primary outcomes and process outcomes (use)

Restate study questions and summarize the answers suggested by the data, starting with primary outcomes and process outcomes (use).

	1	2	3	4	5	
subitem not at all important	○	○	○	○	○	essential

Does your paper address subitem 22-i? *

Copy and paste relevant sections from the manuscript (include quotes in quotation marks "like this" to indicate direct quotes from your manuscript), or elaborate on this item by providing additional information not in the ms, or briefly explain why the item is not applicable/relevant for your study

"This large-scale, nationwide, single-blind, parallel-group randomized controlled trial demonstrated the effectiveness of short, animated storytelling (SAS) videos, using humor and analogy as storytelling techniques, for reducing influenza vaccine hesitancy among a diverse adult population in China. This specialized approach to entertainment-education significantly reduced vaccine hesitancy, highlighting the potential of culturally accessible, wordless SAS videos to support scalable public health messaging. Furthermore, the observed decrease in influenza vaccine hesitancy achieved through SAS narratives built in context of COVID-19 underscores the potential synergies of pro-vaccination messages to boost vaccine uptake across different vaccines."

22-ii) Highlight unanswered new questions, suggest future research

Highlight unanswered new questions, suggest future research.

	1	2	3	4	5	
subitem not at all important	○	○	○	○	○	essential

Does your paper address subitem 22-ii?

Copy and paste relevant sections from the manuscript (include quotes in quotation marks "like this" to indicate direct quotes from your manuscript), or elaborate on this item by providing additional information not in the ms, or briefly explain why the item is not applicable/relevant for your study

您的回答

20) Trial limitations, addressing sources of potential bias, imprecision, and, if relevant, multiplicity of analyses

20-i) Typical limitations in ehealth trials

Typical limitations in ehealth trials: Participants in ehealth trials are rarely blinded. Ehealth trials often look at a multiplicity of outcomes, increasing risk for a Type I error. Discuss biases due to non-use of the intervention/usability issues, biases through informed consent procedures, unexpected events.

	1	2	3	4	5	
subitem not at all important	○	○	○	○	○	essential

Does your paper address subitem 20-i? *

Copy and paste relevant sections from the manuscript (include quotes in quotation marks "like this" to indicate direct quotes from your manuscript), or elaborate on this item by providing additional information not in the ms, or briefly explain why the item is not applicable/relevant for your study

" On the other hand, our study has several limitations. The reliance on self-reported data introduces potential biases, as participants may underreport or overreport their vaccine hesitancy due to social desirability or recall biases. Conducting the intervention and data collection online poses additional limitations, including issues of digital access and engagement. Participants without reliable internet access or those less comfortable with digital platforms may be underrepresented, potentially skewing the results. Moreover, the differences noted within subgroups should be interpreted with caution due to the small sample sizes in some cases. Lastly, the generalizability of these findings beyond the setting of China may be limited. While the study includes a diverse sample, the cultural and regional differences within China and between other countries could affect the applicability of the results to other populations or settings. These limitations suggest the need for further research to validate the findings across different contexts."

21) Generalisability (external validity, applicability) of the trial findings

NPT: External validity of the trial findings according to the intervention, comparators, patients, and care providers or centers involved in the trial

21-i) Generalizability to other populations

Generalizability to other populations: In particular, discuss generalizability to a general Internet population, outside of a RCT setting, and general patient population, including applicability of the study results for other organizations

	1	2	3	4	5	
subitem not at all important	○	○	○	○	○	essential

Does your paper address subitem 21-i?

Copy and paste relevant sections from the manuscript (include quotes in quotation marks "like this" to indicate direct quotes from your manuscript), or elaborate on this item by providing additional information not in the ms, or briefly explain why the item is not applicable/relevant for your study

您的回答

21-ii) Discuss if there were elements in the RCT that would be different in a routine application setting

Discuss if there were elements in the RCT that would be different in a routine application setting (e.g., prompts/reminders, more human involvement, training sessions or other co-interventions) and what impact the omission of these elements could have on use, adoption, or outcomes if the intervention is applied outside of a RCT setting.

	1	2	3	4	5	
subitem not at all important	○	○	○	○	○	essential

Does your paper address subitem 21-ii?

Copy and paste relevant sections from the manuscript (include quotes in quotation marks "like this" to indicate direct quotes from your manuscript), or elaborate on this item by providing additional information not in the ms, or briefly explain why the item is not applicable/relevant for your study

您的回答

OTHER INFORMATION

23) Registration number and name of trial registry

Does your paper address CONSORT subitem 23? *

Copy and paste relevant sections from the manuscript (include quotes in quotation marks "like this" to indicate direct quotes from your manuscript), or elaborate on this item by providing additional information not in the ms, or briefly explain why the item is not applicable/relevant for your study

"Trial Registration:
German Clinical Trials Register; DRKS00024505;
https://drks.de/search/en/trial/DRKS00024505"

24) Where the full trial protocol can be accessed, if available

Does your paper address CONSORT subitem 24? *

Cite a Multimedia Appendix, other reference, or copy and paste relevant sections from the manuscript (include quotes in quotation marks "like this" to indicate direct quotes from your manuscript), or elaborate on this item by providing additional information not in the ms, or briefly explain why the item is not applicable/relevant for your study

"Chen S, Forster S, Yang J, et al. Animated, video entertainment-education to improve vaccine confidence globally during the COVID-19 pandemic: an online randomized controlled experiment with 24,000 participants. Trials 2022; 23(1): 161."

25) Sources of funding and other support (such as supply of drugs), role of funders

Does your paper address CONSORT subitem 25? *

Copy and paste relevant sections from the manuscript (include quotes in quotation marks "like this" to indicate direct quotes from your manuscript), or elaborate on this item by providing additional information not in the ms, or briefly explain why the item is not applicable/relevant for your study

"Research reported in this publication was supported by the Chinese Academy of Medical Sciences & Peking Union Medical College under Project Number 2024-CFT-QT-034, and Horizon Europe (HORIZON-MSCA-2021-SE-01) (Project 101086139 — PoPMeD-SuSDeV). The content is solely the responsibility of the authors and does not necessarily represent the official views of the funding agencies. The study funder had no role in study design; collection, management, analysis, and interpretation of data; writing of the report; nor the decision to submit the report for publication, or authority over any of these activities."

X27) Conflicts of Interest (not a CONSORT item)

X27-i) State the relation of the study team towards the system being evaluated

In addition to the usual declaration of interests (financial or otherwise), also state the relation of the study team towards the system being evaluated, i.e., state if the authors/evaluators are distinct from or identical with the developers/sponsors of the intervention.

	1	2	3	4	5	
subitem not at all important	○	○	○	○	○	essential

Does your paper address subitem X27-i?

Copy and paste relevant sections from the manuscript (include quotes in quotation marks "like this" to indicate direct quotes from your manuscript), or elaborate on this item by providing additional information not in the ms, or briefly explain why the item is not applicable/relevant for your study

您的回答

About the CONSORT EHEALTH checklist

As a result of using this checklist, did you make changes in your manuscript? *

- ○ yes, major changes
- ○ yes, minor changes
- ◉ no

What were the most important changes you made as a result of using this checklist?

您的回答

How much time did you spend on going through the checklist INCLUDING making changes in your manuscript *

I spent approximately three hours reviewing the checklist and making necessary changes to my manuscript.

As a result of using this checklist, do you think your manuscript has improved? *

- (x) yes
- ( ) no
- ( ) 其他:

Would you like to become involved in the CONSORT EHEALTH group?

This would involve for example becoming involved in participating in a workshop and writing an "Explanation and Elaboration" document

- ( ) yes
- ( ) no
- ( ) 其他:

Any other comments or questions on CONSORT EHEALTH

您的回答

STOP - Save this form as PDF before you click submit

To generate a record that you filled in this form, we recommend to generate a PDF of this page (on a Mac, simply select "print" and then select "print as PDF") before you submit it.

When you submit your (revised) paper to JMIR, please upload the PDF as supplementary file.

Don't worry if some text in the textboxes is cut off, as we still have the complete information in our database. Thank you!

Final step: Click submit !

Click submit so we have your answers in our database!

提交

清除表单内容

切勿通过 Google 表单提交密码。

此内容不是由 Google 所创建，Google 不对其作任何担保。 举报滥用行为 - 服务条款 - 隐私权政策

Google 表单
